# Changes in cystic fibrosis transmembrane conductance regulator protein expression prior to and during elexacaftor-tezacaftor-ivacaftor therapy

**DOI:** 10.3389/fphar.2023.1114584

**Published:** 2023-01-27

**Authors:** Frauke Stanke, Sophia T. Pallenberg, Stephanie Tamm, Silke Hedtfeld, Ella M. Eichhorn, Rebecca Minso, Gesine Hansen, Tobias Welte, Annette Sauer-Heilborn, Felix C. Ringshausen, Sibylle Junge, Burkhard Tümmler, Anna-Maria Dittrich

**Affiliations:** ^1^ Department of Pediatric Pneumology, Allergology and Neonatology, Hannover Medical School, Hannover, Germany; ^2^ Biomedical Research in Endstage and Obstructive Lung Disease Hannover (BREATH), German Center for Lung Research, Hannover Medical School, Hannover, Germany; ^3^ Department of Respiratory Medicine, Hannover Medical School, Hannover, Germany

**Keywords:** elexcaftor-tezacaftor-ivacaftor, TRIKAFTA, CFTR protein expression, CFTR glycosylation, CFTR small molecule therapeutics

## Abstract

**Background:** Defects in expression, maturation or function of the epithelial membrane glycoprotein CFTR are causative for the progressive disease cystic fibrosis. Recently, molecular therapeutics that improve CFTR maturation and functional defects have been approved. We aimed to verify whether we could detect an improvement of CFTR protein expression and maturation by triple therapy with elexacaftor-tezacaftor-ivacaftor (ELX/TEZ/IVA).

**Methods:** Rectal suction biopsies of 21 p.Phe508del homozygous or compound heterozygous CF patients obtained pre- and during treatment with ELX/TEZ/IVA were analyzed by CFTR Western blot that was optimized to distinguish CFTR glycoisoforms.

**Findings:** CFTR western immunoblot analysis revealed that—compared to baseline—the levels of CFTR protein increased by at least twofold in eight out of 12 patients upon treatment with ELX/TEZ/IVA compared to baseline (*p* < 0.02). However, polydispersity of the mutant CFTR protein was lower than that of the fully glycosylated wild type CFTR Golgi isoform, indicating an incompletely glycosylated p.Phe508el CFTR protein isoform C* in patients with CF which persists after ELX/TEZ/IVA treatment.

**Interpretation:** Treatment with ELX/TEZ/IVA increased protein expression by facilitating the posttranslational processing of mutant CFTR but apparently did not succeed in generating the polydisperse spectrum of N-linked oligosaccharides that is characteristic for the wild type CFTR band C glycoisoform. Our results caution that the lower amounts or immature glycosylation of the C* glycoisoform observed in patients’ biomaterial might not translate to fully restored function of mutant CFTR necessary for long-term provision of clinical benefit.

## 1 Introduction

Cystic fibrosis (CF) is a life-shortening autosomal recessive trait of exocrine glands and CFTR-expressing epithelia that is caused by mutations in the *Cystic Fibrosis Transmembrane Conductance Regulator* (*CFTR*) gene (Elborn, 2016). The basic defect manifests in impaired chloride and bicarbonate transport across apical epithelial membranes of several organ systems (Elborn, 2016). The most frequent *CFTR* mutation is a 3-bp in-frame deletion, p.Phe508del, that affects the post-translational processing and trafficking and the half-life and function of the ion channel in the plasma membrane (Ameen et al., 2007).

Wild-type CFTR is synthesized in the endoplasmic reticulum (ER) as a mannose-rich glycoisoform (band B) that is converted in the Golgi-apparatus predominantly to the mature complex-glycosylated isoform (band C) (McClure et al., 2016). This maturation process is uneconomical as about 75% of wild-type CFTR is degraded by the ER-associated degradation pathway ERAD (Ward and Kopito, 1994). Maturation of mutant p.Phe508del CFTR is even more ineffective as the newly synthesized p.Phe508del CFTR fails to adopt a wild-type fold in the ER, is targeted to ER-associated degradation (Ward and Kopito, 1994) and is removed faster from the apical membrane by endocytosis (Lukacs et al., 1993). Consequently, p.Phe508del homozygous subjects express only low amounts of complex-glycosylated p.Phe508del CFTR and low or no residual p.Phe508del CFTR-mediated chloride secretory activity in the rectal mucosa, albeit studies of patient’s tissue have confirmed that p.Phe508del-CFTR can be processed to reach the apical membrane, (Dray-Charier et al., 1999; Kälin et al., 1999; Kreda et al., 2005; van Meegen et al., 2013), can be complex glycosylated (Dray-Charier et al., 1999; van Barneveld et al., 2010) and can transport chloride (Bronsveld et al., 2001; Sermet-Gaudelus et al., 2002).

CFTR, like other membrane glycoproteins, has to undergo glycosylation by one or more out of 14 pathways that together rely on a total of 173 glycosyltransferases (Schjoldager et al., 2020). The non-glycosylated polypeptide chain of 1,480 amino acids can only be visualized by western-blotting from biomaterial when deglycosylation enzymes are used (Sarkadi et al., 1992; Kälin et al., 1999). The immature core-glycosylated glycoisoform B (CFTR-B) and the mature complex glycosylated glycoisoform C (CFTR-C) were observed in epithelial model cell lines that express CFTR endogenously (Varga et al., 2004), in various heterologous expression systems (Sarkadi et al., 1992; Ward and Kopito, 1994; Varga et al., 2004; Rowe et al., 2010) and human tissues such as gallbladder (Dray-Charier et al., 1999), colon (van Barneveld et al., 2010), ileum, jejunum and duodenum (Kälin et al., 1999). These mature fully glycosylated CFTR glycoisoforms C differ in size when comparing different sources (Ernst et al., 1994; Kälin et al., 1999; Varga et al., 2004). Moreover, even from one source, a complex glycosylated membrane protein like CFTR-C is not a single entity but a set of molecules that differ in structure and composition of terminal glycosylation residues (McClure et al., 2016), molecular shape and thus, its signal displays a slightly diffuse distribution when analyzed by polyacrylamide gel electrophoresis (Sarkadi et al., 1992; Ernst et al., 1994; Ward and Kopito, 1994; Dray-Charier et al., 1999; Kälin et al., 1999; Varga et al., 2004; Rowe et al., 2010; van Barneveld et al., 2010).

So far, conventional treatment of CF has been supportive, targeting downstream clinical manifestations that result from the loss of CFTR activity. These therapeutic measures improved morbidity and survival, but confer a high burden of care. Recently however, academia and industry have thus focused on the development of small molecule CFTR modulators that restore function of mutant CFTR. These approaches have made CF the first successful example of customized drug development for mutation-specific therapy whose effects therefore have implications beyond CF. Both US and European regulatory agencies (FDA and EMA) have recently approved the combination of the type III corrector elexacaftor (ELX), the type I corrector tezacaftor (TEZ) and the gating potentiator ivacaftor (IVA) for treatment of patients with CF with at least one p.Phe508del allele (Heijerman et al., 2019; Middleton et al., 2019). Taking the surrogate parameter of the forced expiratory volume in 1 s, FEV1, treatment with ELX/TEZ/IVA improved lung function by 12 percentage points in patients who are homozygous for p.Phe508del and by 14 percentage points in patients who are compound heterozygous for p.Phe508del and a so-called “minimal-function” mutation (Heijerman et al., 2019; Middleton et al., 2019). Sweat chloride concentrations as a measure of the defective CFTR—mediated chloride reabsorption in the sweat duct showed a mean 47–50 mmol/L reduction, thus decreasing to an intermediary or even normal range, suggesting that CFTR function in the sweat duct had been reverted to wild type (Heijerman et al., 2019; Middleton et al., 2019). Prior to ELX/TEZ/IVA treatment, p.Phe508del homozygous patients have received TEZ/IVA as a reference treatment (Heijerman et al., 2019).

The effect of targeted CFTR modulator therapy on CFTR protein expression has been observed during short-term exposure to transfected cell lines where constraints of ER processing and Golgi-associated post-translational glycosylation may differ compared to the effects achieved in patients’ epithelial tissues. Such *in vitro* results suggest that incubation with ELX/TEZ/IVA leads to an increase of mature CFTR (Capurro et al., 2021; Becq et al., 2022) and facilitates clustering of CFTR into lipid rafts and ceramide-rich platforms (Abu-Arish et al., 2022). Closer to the *in vivo* situation are primary epithelia derived from healthy controls or p.Phe508del-CFTR homozygous CF patients in which CFTR function has been assessed with electrophysiology. These studies show that lumacaftor (LUM) (Gentzsch et al., 2017; Pranke et al., 2017) or ELX (Shaughnessy et al., 2021) can increase p.Phe508del-CFTR function in primary epithelia and that ELX/TEZ/IVA corrects p.Phe508del function to about two thirds of wild-type CFTR function (Veit et al., 2020). Western blot analysis of primary airway epithelia shows that p.Phe508del maturation can be partially corrected by LUM (Cholon et al., 2014; Veit et al., 2016) and TEZ (Gentzsch et al., 2021) and interestingly, that IVA can reduce CFTR maturation efficacy in comparison to LUM single molecule treatment (Cholon et al., 2014) or in heterologous cell lines in ELX/TEZ/IVA triple therapy (Becq et al., 2022).

However, data on the degree of *in vivo* correction of CFTR protein expression by ELX/TEZ/IVA—as a highly potent modulator treatment which permits treatment of the majority of affected patients with CF, achieving impressive clinical effects (Heijerman et al., 2019; Middleton et al., 2019)—have remained elusive. Such data could clarify the potential sources of the heterogeneity of the individual clinical response and anticipate long-term efficacy of ELX/TEZ/IVA.

Based on the results in clinical trials, we hypothesized that treatment with ELX/TEZ/IVA promotes maturation and surface expression of p.Phe508del CFTR protein in epithelium. To test this hypothesis, we examined rectal suction biopsies of p.Phe508del homozygous and compound heterozygous CF patients with high-resolution immunoblot analysis prior and during treatment with ELX/TEZ/IVA.

## 2 Patients and methods

### 2.1 Ethics and participants

Results presented are part of a larger, multi-center trial at four study centers of the German Center for Lung Research designed to analyze effects of ELX/TEZ/IVA treatment on different clinical parameters and biomaterials (NCT04732910) (Graeber et al., 2022). Sampling of rectal biopsies for CFTR protein content by Western blot analyses was only performed in the subgroup of patients recruited at the Hannover Medical University site of the study, according to the ethical approval # 8922_BO_S_2020 from the Hannover Ethics Committee.

We obtained written informed consent from all patients included in the study, their parents or legal guardians. Patients were eligible to participate if they were at least 12 years old, homozygous for p.Phe508del-*CFTR* or compound-heterozygous for p.Phe508del-*CFTR* and a minimal function mutation, had no prior exposure to ELX/TEZ/IVA and were willing to remain on a stable medication regimen, including ELX/TEZ/IVA according to the patient labeling and the prescribing information for the duration of study participation. Exclusion criteria were an acute respiratory infection or pulmonary exacerbation at baseline, intranasal medication changes within 14 days prior to baseline and a history of transplantation. In total, 21 patients were included in our analyses of CFTR protein content of rectal biopsies.

### 2.2 Outline of CFTR immunoblot

To optimize the sensitivity and specificity of the immune-chemical CFTR signal, conditions for electrophoresis were selected that preferentially resolve proteins within the range of 100–300 kDa (see Section 2.4 below) and the Western blot was then probed with a mixture of four monoclonal antibodies that are known to detect CFTR epitopes with high affinity and specificity (see Section 2.6 below).

### 2.3 Preparation of lysates from rectal suction biopsies

Two to four rectal suction biopsies were obtained with a rectal suction biopsy tool Model SBT-100 (Trewavis Surgical, Australia) and frozen at −80°C after measurement of intestinal current according to the SOP of the ECFS DNWG, V.2.7. Frozen biopsies were thawed for 10 min at room temperature in 50 µL of SDS-rich lysis buffer (50 mM Tris pH 6.8, 2% (w/v) SDS, 10% (v/v) glycerol, 100 mM dithiothreitol (DTT), 1:50 proteinase inhibitor cocktail (SRE0055-1BO; Sigma Aldrich; MO, United States), 0.2 mM phenylmethylsulfonylfluoride, 1:10,000 Omnicleave endonuclease (OC7850K; Biozym; Germany). Samples were homogenized in a 1.5 mL sample tube with a fitting pistil (pistil PS, Kisker, Steinfurt) whereby care was taken to wedge the biopsy between pistil and sidewall of the tube during homogenization until the tissue was disintegrated with several up-and-down strokes of the pistil. Care was taken to rescue all material left on the surface of the pistil with a pipette tip, next the lysate was incubated at 37°C for 30 min, followed by a second homogenization procedure with several strokes of the pistil. Lysates were next sheared by pipetting the 50 µL volume ten times with a 200 µL pipette-tip. Centrifugation for 10 min at 13,000 rpm (5424R, Eppendorf, Hamburg, Germany) yielded a supernatant of 35–45 µL that was adjusted with the same volume of glycerol. For this study, biomaterials were analyzed after storage at −80°C for 7–14 days.

### 2.4 Gel electrophoresis

Protein content of lysates was semi-quantified with minute aliquots of 1:5; 1:10; 1:30; 1:60 serially diluted in 150 mM NaCl. One µL volumes of the diluted samples were spotted on a Whatman 3 MM paper in comparison to a serial dilution of 5.0 μg/μL to 0.1 μg/μL bovine serum albumin in 150 mM NaCl. Spots were dried, stained in Coomassie solution (0.1% Coomassie brilliant blue in 25% isopropanol, 10% acetic acid) for 10 s, and the stained Whatman paper was thoroughly rinsed using running tap water. Protein concentration of lysates was estimated by comparing staining of spotted samples to the staining of control proteins.

Electrophoresis was carried out in a Mini-PROTEAN Tetra Cell (#165-8001; Bio-Rad Laboratories GmbH; Munich; Germany) using 6% polyacrylamide (PAA; Rotiphorese Gel 30, crosslink 37.5:1; Roth; Karlsruhe, Germany). The separation matrix of 6% PAA was casted to yield a separation distance of 6.5 cm below a very narrow 4% PAA gel. Equal amounts of either baseline and treatment lysate were loaded side-by-side in a total volume of 25 µL each. Sample volumes were adjusted with 50 mM Tris pH 6.8, 2% (w/v) SDS, 50% (w/v) glycerol whereby 100 mM DTT and 1:50 proteinase inhibitor cocktail were freshly added prior to a mild denaturation step of 30 min at 37°C. 16HBE14o-cell lysates were used as a positive control on each gel. Electrophoretic mobility of samples was judged against a prestained molecular weight marker (PageRuler Plus Prestained Protein Ladder; #26619; Thermo Fisher; Darmstadt; Germany). Electrophoresis was carried out at 12 V for approximately 20 h at 4°C whereupon the electrophoresis was continued at 60 V for approximately 3 h until the 72 kDa marker had almost reached the lower edge of the polyacrylamide gel.

### 2.5 Transfer of proteins to membrane

Proteins were transferred to an uncharged supported nitrocellulose membrane (Amersham Protran Supported Nitrocellulose Blotting-Membrane; 0.45 µm pore size; #10600016; VWR; Darmstadt; Germany) by tank blotting in a Mini Trans-Blot Electrophoretic Transfer Cell (#170-3935; Bio-Rad Laboratories GmbH; Munich; Germany). Polyacrylamide gels were mounted into the gel holder cassette whereby the high molecular weight edge of the gel was placed at the cassette’s hinge. Transfer was done in 125 mM Tris, 950 mM glycine, 0.02% (w/v) SDS at 44 mA for approximately 23 h whereby the tank blot apparatus was submerged in ice in a Styrofoam container. Upon completion of tank blot, the polyacrylamide gel was stained with Coomassie to visualize non-transferred high molecular weight proteins.

### 2.6 Serial detection of CFTR and vinculin

Membranes were vertically cut between the lane containing the molecular weight marker and the adjacent samples which were separately processed.

Positions of prestained molecular weight marker bands were marked with a ballpoint pen. Next, the marker lane membrane was incubated in 0.05% (v/v) Tween20 in 140 mM NaCl, 2.7 mM KCl, 16 mM Tris, pH 7.4 (TBS) for 1 h, next for 1 h in secondary antibody solution (1:30,000 goat anti-mouse IgG (ab97040; Abcam; Cambridge; United Kingdom) in StartingBlock (#37542; Thermo Fisher; Darmstadt; Germany) with 0.1% Tween20) whereupon signals of the molecular weight marker could be visualized with horse-radish peroxidase substrates (see below).

The membranes of biopsy lysates were incubated in StartingBlock with 0.1% Tween20 for 1 h. Incubation with primary antibodies was carried out in a float-your-blot set-up: parafilm was placed in a 12 cm square petri dish using a small volume of 0.05% Tween20 in TBS between the plastic surface and the parafilm. Next, 1,000 µL of primary antibody solution (see below) were distributed onto the parafilm in a line parallel to the parafilm’s edge. Next, the membrane was positioned at an angle over the parafilm whereby the side containing the proteins was oriented towards the parafilm and the edge containing the high molecular weight proteins was aligned with the primary antibody solution. To incubate the entire membrane surface with the small volume of primary antibody solution, the membrane was slowly lowered towards the parafilm starting at the edge with the high molecular weight proteins and continuing towards the edge where lower molecular weight proteins were located. The petri dish was covered with its lid and the entire assembly was placed in a container with 0.05% Tween20 in TBS to prevent evaporation. Primary antibodies were incubated for 18 h at 4°C.

Immune-reactive signals of biopsy lysates were generated by sequential probing with antibodies: 1st detection of CFTR (1st antibody: equimolar mix of CFTR-AK 596 + 570 + 217 + 660 (Cystic Fibrosis Foundation CFTR Antibody Distribution program; Chapel Hill; NC, United States) diluted 1:400 in StartingBlock with 0.1% Tween20; 2nd antibody: goat anti-mouse IgG (ab97040; Abcam; Cambridge; United Kingdom) diluted 1:7500 in StartingBlock with 0.1% Tween20); 2nd detection of CFTR (see above); stripping three times for 10 min each with 200 mM glycine, 0.1% SDS, 2% Tween20, pH 2.2; detection of vinculin (1st antibody: anti-vinculin antibody (ab130007; Abcam; Cambridge; United Kingdom) diluted 1:500 in StartingBlock with 0.1% Tween20; 2nd antibody: goat anti-mouse IgG (ab97040; Abcam; Cambridge; United Kingdom) diluted 1:7500 in StartingBlock with 0.1% Tween20).

### 2.7 Development of CFTR and vinculin signals with HRP substrates and densitometry

Membranes with biopsy lysates were incubated sequentially with SuperSignal West Pico (34078; Thermo Fisher; Darmstadt; Germany) and SuperSignal West Femto Max. Sensitivity (34096; Thermo Fisher; Darmstadt; Germany). To ensure that signals in all lanes were visualized, exposure times were varied between 3 s and 30 min for PICO and next, between 3 s and 30 min for FEMTO yielding about 15 different exposures of each primary antibody target. Scans were acquired on a DNR-MF-ChemiBIS 3.2 Bio-Imaging System (Berthold Technologies, Bad Wildbad, Germany). For densitometry, an exposure with high signal intensity was selected, avoiding saturation of corresponding baseline and treatment pairs. Densitometry of digitized scans was performed with GelAnalyzer 19.1 (www.gelanalyzer.com by Istvan Lazar Jr., PhD and Istvan Lazar Sr., PhD, CSc). Datasets of CFTR isoforms were compared from paired samples obtained from specimens prior to and after the start of therapy with ELX/TEZ/IVA and the effect of ELX/TEZ/IVA was judged by Wilcoxon-signed rank test.

### 2.8 Censoring criteria for semi-quantification of signals generated by Western-blot

As biopsy lysates can contain mucus proteins that are detected by protein quantification of the primary lysates but do not migrate into the polyacrylamide gel matrix, we used Coomassie staining after transfer onto the membrane to ensure that the amount of proteins that have entered the gel matrix are comparable between reference or ELX/TEZ/IVA-treated sample of one patient. Furthermore, signals for epithelial vinculin were used to judge whether paired reference and ELX/TEZ/IVA-treated biomaterials were comparable with respect to the proportion sampled of epithelial tissue. If either of these two measures showed a difference between biomaterials, paired samples were excluded from semi-quantitative analysis (see Supplementary Figures S2A–L).

In detail, we have obtained CFTR Immunoblots prior to and after the start of ELX/TEZ/IVA treatment from rectal suction biopsies of 21 patients. We have judged paired biomaterials by two controls: Firstly, based on non-specific protein staining using Coomassie of leftover material after transfer of whole-cell lysates to the membrane, we have verified that the amount of proteins capable to enter the polyacrylamide matrix is comparable between samples obtained pre-treatment and after ELX/TEZ/IVA treatment from one patient. Next, specific vinculin staining was used to judge whether the paired biopsy samples contain an equivalent amount of epithelium. Based on Coomassie and/or specific vinculin staining, we excluded eight samples from further densitometric analysis of CFTR band C* since samples prior-to-treatment and corresponding ELX/TEZ/IVA samples were incomparable for eight pairs (N°2, N°7, N°8, N°11, N°12, N°17, N°18, and N°21). Densitometry confirms that censored samples differed stronger than samples included for densitometry: Signals for vinculin and metavinculin of censored eight sample pairs differed by 1.3 fold (SD 1.6 fold) when comparing prior-to-treatment and ELX/TEZ/IVA-sample. Vinculin and metavinculin signals in 14 sample pairs accepted for densitometry differed by 0.4 fold (SD 0.2 fold) when comparing prior-to-treatment and ELX/TEZ/IVA lane.

For the remaining 14 samples, CFTR band C* was quantified by densitometry from both paired samples. If possible, we have evaluated more than one CFTR detection as indicated in Supplementary Figures S2A–L. Minute signals for either the sample obtained pre-treatment or for the sample obtained after ELX/TEZ/IVA-treatment were observed in five sample pairs (N°4, N°6, N°9, N°10, and N°13) which were resolved as follows: The intensity of CFTR band C* was minute in four control samples (N°6, N°9, N°10, and N°13), making normalization to 100% as expression level prior to start of treatment error prone. Based on obtained primary data of samples N°19 and N°20 corresponding to an increase of CFTR C* of 400%, 274% and 405%, we cautiously used a cut-off value of >300% gain in signal for band CFTR C* to describe the increase in CFTR expression in these samples. In sample pair N°4, the low intensity band C* could not be quantified in the sample obtained after treatment with ELX/TEZ/IVA and thus decrease upon treatment was not quantified in this sample pair.

In summary, we have obtained semi-quantitative data for the change in CFTR-C* expression upon treatment with ELX/TEZ/IVA for 13 out of 21 paired biosamples (see Figure 2C, source data shown in Supplementary Figure S2).

### 2.9 Statistics

Differences in CFTR glycoisoform expression comparing rectal suction biopsies obtained prior to treatment and patient tissue obtained under ELX/TEZ/IVA-treatment were judged by the non-parametric Wilcoxon signed-rank test. Critical values for small number of observations were judged based on: S. Siegel, Non-parametric Statistics, McGraw Hill Book Comp., London 1956, p.254 as described in E. Weber, Grundriss der biologischen Statistik, VEB Gustav Fischer Verlag, Jena 1986.

Korrelation of CFTR protein expression and clinical parameters (Supplementary Figure S3) was judged by Spearman’s rank correlation coefficient with correction for ties within the data set.

## 3 Results

### 3.1 Design of the study and clinical outcome

Fifteen female and six male patients with CF participated in the study (Table 1). We obtained anthropometry, spirometry, sweat chloride concentrations and rectal suction biopsies for CFTR immunoblot analysis at the day prior to the first administration of ELX/TEZ/IVA and after 12–18 weeks (median: 16 weeks) of continuous triple therapy. Six patients were p.Phe508del homozygous and fifteen patients were p.Phe508del heterozygous in combination with a minimal function *CFTR* mutation. Twenty participants were naïve for CFTR modulation at the start of the triple therapy, only the oldest participant had been on regular combination treatment with tezacaftor/ivacaftor until the day of first assessment.

**TABLE 1:** Study participants.

Patient No.^a^	*CFTR* genotype (Legacy name)	Gender	Assessment prior to ELX/TEZ/IVA therapy				Assessment during ELX/TEZ/IVA therapy			
			age (yr)	BMI (kg/m^2^)	FEV1 %pred	Sweat Cl^−^ (mM)	age (yr)	BMI (kg/m^2^)	FEV1 %pred	Sweat Cl^−^ (mM)
1	F508del/2184insA	F	16.0	21.9	93	106	16.3	22.5	106	20
2	F508del/CFTRdele2,3 (21 kb)	F	21.3	19.2	53	103	21.6	19.2	82	14
3	F508del/2721del11	F	14.0	17.4	85	104	14.3	18.6	94	25
4	F508del/CFTRdele2,3 (21 kb)	F	13.7	20.0	129	104	14.1	19.6	147	54
5	F508del/N1303K	F	17.3	25.0	71	109	17.6	25.7	83	101
6	F508del/CFTRdele2,3 (21 kb)	F	13.8	19.4	110	110	14.0	22.9	126	40
7	F508del/F508del	F	12.5	15.6	53	95	12.8	17.1	89	10
8	F508del/2184delA	F	14.7	18.5	104	113	15.0	18.8	115	65
9	F508del/F508del	F	13.6	19.8	107	104	13.9	19.8	112	27
10	F508del/1078delT	F	12.7	17.9	101	88	13.0	18.6	117	41
11	F508del/G542X	F	20.7	15.8	46	92	21.2	18.1	76	38
12	F508del/394delTT	F	12.0	14.2	63	98	12.4	16.5	83	70
13	F508del/F508del	F	24.3	20.2	93	101	24.6	21.3	124	79
14	F508del/1078delT	F	15.5	17.9	113	99	15.8	17.9	126	44
15	F508del/F508del	F	14.2	15.2	74	87	14.5	16.9	103	25
16	F508del/G542X	M	17.1	20.2	84	108	17.5	20.3	115	90
17	F508del/R553X	M	44.0	28.1	82	102	44.4	29.5	87	53
18	F508del/E822X	M	12.8	21.1	80	108	13.2	21.1	88	36
19^b^	F508del/F508del	M	44.8	21.1	62	108	45.2	22.5	88	52
20	F508del/F508del	M	13.9	15.8	95	98	14.2	16.1	116	30
21	F508del/2184delA	M	12.7	15.1	89	115	13.1	16.8	118	50
Median [IQR]			14.2 [13.6–17.3]	19.1 [15.8–20.2]	85 [71–100]	104 [98–108]	14.5 [13.9–17.6]	19.2 [17.9–21.3]	106 [88–117]	41 [27–54]

^a^ Results presented are part of a larger, multi-center trial at four study centers of the German Center for Lung Research designed to analyze effects of ELX/TEZ/IVA treatment on different clinical parameters and biomaterials (NCT04732910) (Graeber et al., 2022). Sampling of rectal biopsies for CFTR protein content by western blot analyses was only performed in the subgroup of patients recruited at Hannover Medical School, according to the ethical approval # 8922_BO_S_2020 from the Hannover ethics committee.

^b^ Patient 19 had continuously administered tezacaftor/ivacaftor for 18 months prior to triple therapy. All other study participants were modulator–naïve at baseline.

Consistent with the outcome of the phase III trials, the study participants who had been naïve for CFTR modulation improved anthropometry and lung function (Table 1). Chloride concentration in sweat test was in the pathological range at baseline (median 104 mmol/L, range 87–115 mmol/L) and decreased during exposure to ELX/TEZ/IVA by a mean of 56 mmol/L to a median sweat chloride concentration of 41 mmol/L (range 10–101 mmol/L). Sweat chloride decreased to the normal and intermediary levels in six and ten subjects, respectively, but remained in the pathological range in five subjects (Table 1).

### 3.2 CFTR immunoblot analysis of rectal biopsies from CF patients and healthy controls

**FIGURE 1:**
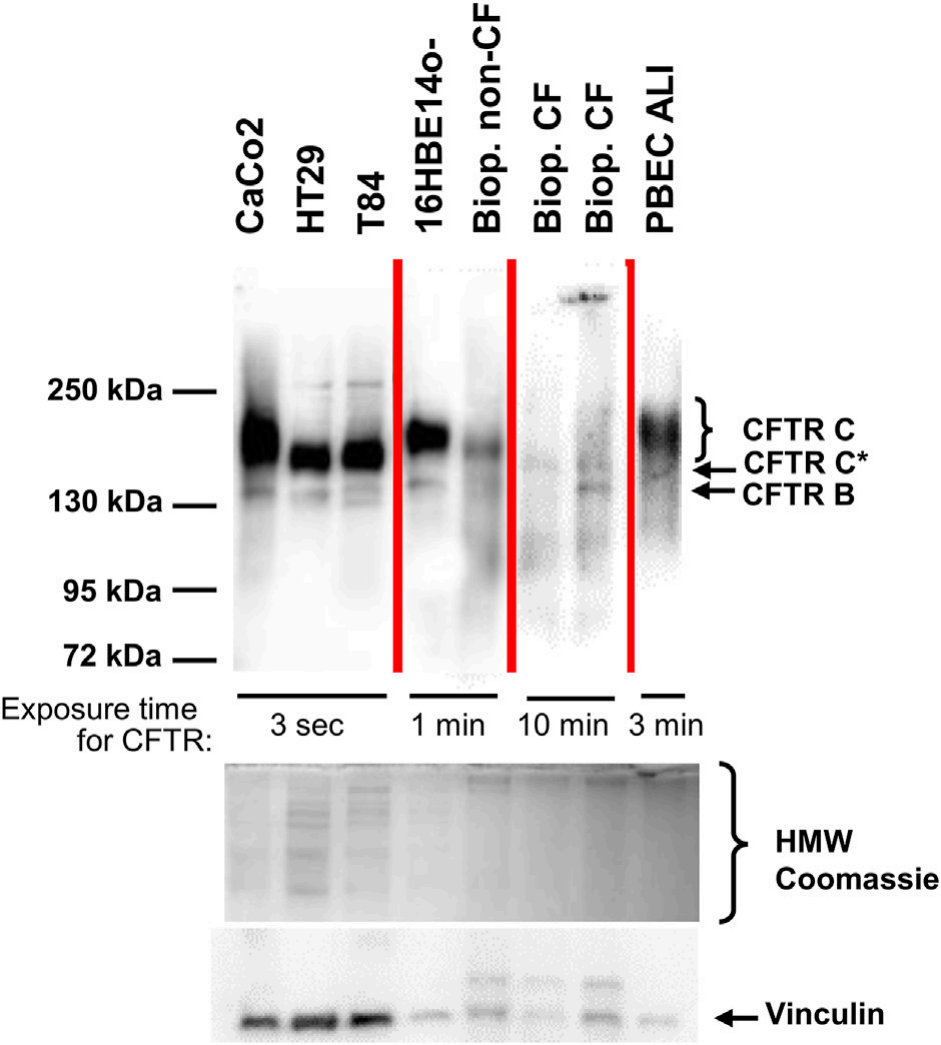
CFTR immunoblot analyses of epithelial cell lines, primary bronchial epithelium and from rectal biopsies from one healthy control and two CF samples. CFTR signals were obtained from the intestinal cancer cell lines CaCo2, HT29, T84, from the immortalized respiratory epithelial cell line 16HBE14o-, from primary peripheral bronchial epithelium (PBEC) generated by air-liquid interface (ALI) culture and compared to a rectal suction biopsy derived from a non-CF control (“Biop. non-CF”) and from two CF rectal suction biopsies (“Biop. CF”). Western blots reveal a narrow band B at 130 kDa and a much broader band C between 150 and 180 kDa in all samples except for the CF patients’ biopsies. Rectal biopsies from CF patients showed a band B similar to the other samples and generated a narrow signal, migrating slower than band B at approximately 140 kDa designated as CFTR C*. A poly-disperse wild-type band C visible in all other non-CF samples was absent from CF biopsies. Samples were loaded on one gel, yet this figure is a composite as visualization of signals was done using exposure times between 3 s and 10 min for CFTR as noted within the figure. Source data of all four exposures of the entire membrane is provided in the supplement. Protein amount loaded was as follows: CaCo2, HT29, T84, 16HBE14o-: 30 μg; PBEC ALI: 25 μg; rectal suction biopsies: 75 µg.

Figure 1 compares the gel-separated CFTR immunochemical signals of intestinal and pulmonary reference cell lines commonly used in CFTR research, primary bronchial epithelial cells (PBEC) grown under air-liquid interface (ALI) condition, a rectal suction biopsy from a non-CF control and two samples from CF rectal suction biopsies. Non-CF samples all displayed a narrow band B at 130 kDa and a much broader band C between 150 and 180 kDa. Yet, wild-type CFTR band C from CaCo2, 16HBE14o- and from PBEC ALI migrated slower than wild-type CFTR from HT29 and T84 or the non-CF biopsy, confirming size and/or glycosylation differences between wild-type CFTR of different origins (Ward and Kopito, 1994; Kälin et al., 1999; McClure et al., 2016). The rectal mucosa of the healthy control shares the position, width and intensity of the CFTR immune-reactive signals of a faint band B and a strong band C at the previously published positions of 150–180 kDa (complex glycosylated wild-type CFTR-C) (Ward and Kopito, 1994; Kälin et al., 1999; McClure et al., 2016) and 130 kDa (core-glycosylated CFTR-B) (Ward and Kopito, 1994; Kälin et al., 1999; McClure et al., 2016) with those of the intestinal cell lines T84 and HT29 (Figure 1).

**FIGURE 2:**
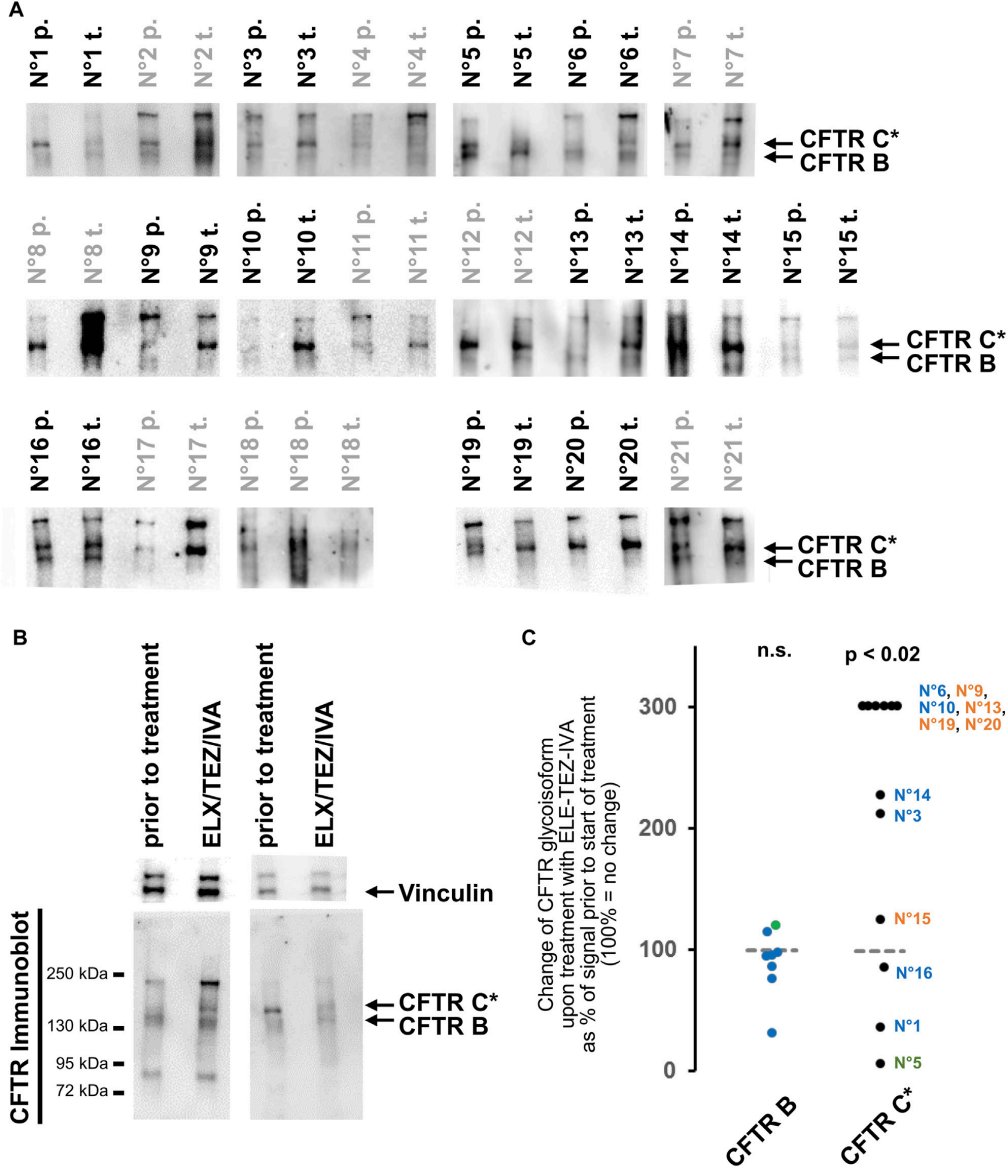
Changes in CFTR glycoisoforms from rectal suction biopsies prior to and after initiation of treatment with ELX/TEZ/IVA. **(A)** CFTR immunoblot signals of rectal biopsy lysate pairs from 21 patients. Samples prior-to-treatment (labelled “p.”) and samples obtained after ELX/TEZ/IVA treatment (labelled “t.”) were incomparable as judged by Coomassie staining and by vinculin detection for these eight pairs: N°2, N°7, N°8, N°11, N°12, N°17, N°18, N°21 (labelled in grey; see methods section for details on censoring). Exposures are selected to visualize band C* and B in CF samples. Calibration of C* vs. CFTR-C from 16HBE14o- is shown as source data of the entire patient cohort including Coomassie staining of high molecular weight proteins and vinculin signals in Supplementary Figure S2. **(B)** Western blot of two representative examples. Left sample pair shows an increase of CFTR C* upon treatment with ELX/TEZ/IVA (patient N°6) while right sample pair shows a decrease of CFTR* upon treatment with TEZ-ELE-IVA (patient N°1). Additionally, the left sample pair shows the <95 kDa and 240 kDa immune-reactive signals visible in about a third (<95 kDa) and all but one (240 kDa) samples. **(C)** Densitometry was carried out on side-by-side loaded lanes of pre- and post-treatment samples for 12 out of 21 biopsy lysate pairs that yielded comparable signals for whole protein content and vinculin [see Panel **(A)** and Section 2 for details on censoring]. An increase of CFTR C* is observed (*p* < 0.02, Wilcoxon signed rank test) while the signal intensity of the core glycosylated band B is unaltered by ELX/TEZ/IVA. The patient’s CFTR mutation genotype is color-coded as follows: p.Phe508del homozygotes—terracotta (N°15, N°19, N°20, N°13, N°9); p.Phe508del compound heterozygotes with a class I mutation—blue (N°1, N°16, N°3, N°14, N°10, N°6); p.Phe508del/N1303K compound heterozygote—green (N°5).

By applying the same experimental conditions, CFTR immune-reactive signals of the selected CF rectal biopsies are barely detectable, even at significantly longer exposure times indicating that the intestinal epithelia of CF patients express only low amounts of mutant CFTR (Figure 1). Even more important, side-by-side detection of CFTR from the non-CF control and from CF biopsies revealed that the signal of the glycosylated isoform obtained from CF biopsies is detected at a lower molecular weight, or alternatively, exhibits a more compact three-dimensional structure to allow faster migration through the polyacrylamide matrix. Twenty out of twenty-one biopsies obtained prior to start of ELX/TEZ/IVA-treatment displayed such a mutant CFTR C* signal and the typical shape of complex glycosylated wild-type CFTR band C was absent in all analyzed CF biopsies (Figure 2A; Supplementary Figure S2). We have named this differently glycosylated form of mutant CFTR, typical for CF patients’ rectal biopsies, “band C*” to denote that its electrophorectic mobility is not equivalent to complex glycosylated wild-type CFTR band C.

### 3.3 Changes in CFTR expression patterns after initiation of ELX/TEZ/IVA treatment

We next addressed CFTR glycoisoform patterns 8–16 weeks after initiation of treatment with ELX/TEZ/IVA. To this end, we analyzed rectal suction biopsies obtained from the study participant prior to and during ELX/TEZ/IVA-treatment side-by-side by semi-quantitative immune-detection of CFTR. In 16 out of 21 lysates obtained prior to start of ELX/TEZ/IVA-treatment, the core-glycosylated isoform CFTR B at 130 kDa was seen. In all but one sample prior to and in all 21 samples obtained after ELX/TEZ/IVA-treatment, CFTR band C* was the dominant CFTR glycoisoform in CF rectal suction biopsies (Figure 2A; Supplementary Figures S2A–L). Additionally, a low molecular weight band below 95 kDa in size could be observed in about a third and a high molecular weight band of approximately 240 kDa in size was seen in 18 out of 21 sample pairs. If present, the <95 kDa band and/or the 240 kDa band appeared in patient samples obtained at baseline and during ELX/TEZ/IVA treatment (Figure 2B, two left lanes and Supplementary Figures S2A–L).

Since the glycosylated CFTR band C* was the most prominent CFTR glycoisoform in most analyzed CF rectal suction biopsies, we aimed to quantify the change of CFTR C* induced by ELX/TEZ/IVA treatment (Figure 2C). In the majority of sample pairs an increase of CFTR C* signal intensity was seen in biopsies taken after the onset of ELX/TEZ/IVA-treatment (Figure 2B, two left lanes). In eight out of 12 sample pairs that were eligible for semi-quantification of CFTR signals (see methods for details), the signal for C* was at least twofold higher during triple therapy than at baseline (Figure 2C, *p* < 0.02, Wilcoxon signed rank test). However, in two out of 12 samples we observed a decrease in CFTR C* signal upon treatment to about half of that of the baseline sample (Figure 2B, two right lanes; Figure 2C). Of note, the strongest decline in CFTR-C* was noted in patient N°5 who is compound heterozygous for p.Phe508del and the missense variant N1303K. All other participants are either homozygous for p.Phe508del or compound heterozygous p.Phe508del with a stop or frameshift mutation, whereby no functional CFTR protein is expected from the latter class I mutations. In other words, biomaterial from the one compound heterozygous study participant who carries two, not one, class II alleles that could be improved by ELX/TEZ/IVA displayed most loss of CFTR-C* protein expression. Furthermore, absence or presence of CFTR-B and change in CFTR-C* expression was CFTR genotype-dependent among 12 cases eligible for densitometry as judged by comparable vinculin signals in samples taken prior to and after start of ELX/TEZ/IVA therapy allowed quantification of CFTR signals. Signals for CFTR-B were absent or too low for comparative quantification in biosamples from five p.Phe508del homozygotes while among seven compound heterozygotes, CFTR-B could be quantified but did not change upon ELX/TEZ/IVA therapy (Figure 2C). In contrast, increase of CFTR-C* was higher in p.Phe508del homozygotes compared to compound heterozygotes who carried only one allele: four out of five p.Phe508del homozygotes showed an increase in CFTR-C* of at least 300% while for five out of seven compound heterozygotes—carrying one p.Phe508del allele only—less than 300% increase in CFTR-C* was observed. A decrease in CFTR-C* was only seen in samples from three compound heterozygous patients.

We carefully interrogated the immunoblots for a CFTR immune-reactive signal with migratory properties intermediate between the mutant glycosylated CFTR band C* and the mature, wild-type CFTR band C seen in the 16HBE14o-control samples. We observed such a band CFTR C** in samples from three patients (Supplementary Figure S2M). However, it was a faint signal and distinguishable from wild-type CFTR band C by two criteria, i.e., a higher electrophoretic mobility and lower width of the immune-reactive signal indicating an altered shape and/or lower polydispersity of the N-glycans. This CFTR C** was visible in two samples at baseline and in one sample during ELX/TEZ/IVA therapy (Supplementary Figure S2M).

## 4 Discussion

Treatment of CF with small molecules to increase cell-surface CFTR expression and thereby protein function is a first successful example of mutation-specific therapy for genetic diseases. Similar approaches to restore functionality of mutated proteins can be envisioned for a large range of genetic diseases where mutations also affect synthesis, post-translational processing and trafficking and thereby protein functions. Insight into the effects of such an approach therefore has the potential to reveal broad implications for the development of, not only CFTR modulators, but also for small-molecule approaches for other genetic diseases.

In that line, our data provide initial evidence that ELX/TEZ/IVA indeed increases CFTR protein expression in rectal suction biopsies in the majority of patients. Interestingly, our results also show that in rectal epithelium of CF patients the molecular weight of expressed CFTR differs between CF patients and non-CF controls suggesting persistent alterations in post-translational glycosylation, which are not affected by ELZ/TEZ/IVA. Thus, our data provide two novel aspects as explanations for the patient-to-patient variability as a possible clue to individual responses (Heijerman et al., 2019; Middleton et al., 2019).

Our data on human rectal suction biopsies collected prior to and after the start of therapy with ELX/TEZ/IVA identified a partially mature complex-glycosylated isoform CFTR C* prior to ELX/TEZ/IVA of varying intensity in all patients. Migratory properties of this band C* differed from fully glycosylated wild-type CFTR band C which we could observe in healthy controls and in intestinal epithelial cell lines and airway epithelial cells lines and primary airway epithelial cells (Figure 1). To our knowledge, our data are the first to identify this CF-typical migratory pattern of the CFTR protein in CFTR modulator naïve or treated patients. Interestingly, Sette et al. (2021) could recently visualize CFTR from patient-derived nasal epithelial conditionally reprogrammed stem cells whereby three p.Phe508del-CFTR homozygotes displayed a CFTR glycoisoform phenotype in ALI culture similar to the C*/B—combination observed in patient’s intestinal epithelium *ex vivo* in our study. Moreover, Dekkers et al. (2016) showed western blot data from intestinal organoids whereby one p.Phe508del homozygote and three p.Phe508del compound heterozygotes display a faint glycosylated CFTR isoform with migratory properties at the lower rim of the wild-type signal, thus in consistency with the band CFTR-C* described in this work. Taken together, these data (Dekkers et al., 2016; Sette et al., 2021, this work), suggest that CFTR-C* represents the *status quo* of p.Phe508del-CFTR expression in CF patients *in vivo*.

Our data upon treatment with ELX/TEZ/IVA suggest that ELX/TEZ/IVA facilitated the posttranslational processing of some mutant CFTR, but apparently did not succeed in generating the poly-disperse spectrum of N-linked oligosaccharides that is characteristic for wild type CFTR band C. Migratory properties observed by polyacrylamide gel electrophoresis of the mutant complex-glycosylated isoform CFTR C* are in between those of the core-glycosylated isoform B and the mature glycoisoform C of wild-type CFTR (Figure 1). This suggests that the repertoire of glycosylation enzymes resident in the mid-to-trans-Golgi compartment (Schjoldager et al., 2020) has not been fully utilized to generate the branched and elongated N-linked oligosaccharides typical of mature wild-type CFTR (McClure et al., 2016).

Previously, western-blot data have confirmed that an increase of mature CFTR is observed upon therapy with ELX/TEZ/IVA (Capurro et al., 2021; Becq et al., 2022). We detected the mannose-rich ER isoform band B in similar amounts in epithelia of non-CF origin and in CF specimens collected at baseline or during triple therapy. In that, our results propose that the biosynthesis and turnover of the p.Phe508del-CFTR ER glycoisoform does not significantly differ from that of the wild-type protein and is not affected by triple combination CFTR modulation. Conversely, the mutant CFTR band C* glycoisoform was enhanced by ELX/TEZ/IVA in 60% of samples by at least twofold, suggesting that an improvement in CFTR processing and maturation in the ER and cis-Golgi beyond the core-glycosylation that shapes the CFTR glycoisoform B is achieved by ELX/TEZ/IVA (Figure 2). Since non-conventional trafficking has been noticed for CFTR (Yoo et al., 2002; Gee et al., 2011), it is conceivable that mutant CFTR C* can reach the plasma membrane of epithelia and function as a chloride- and bicarbonate channel which is corroborated by ELX/TEZ/IVA’s effect on sweat chloride levels and other biomarkers of CFTR function (Heijerman et al., 2019; Middleton et al., 2019; Graeber et al., 2022). Notably however, the change in mutant CFTR C* induced by ELX/TEZ/IVA observed *ex vivo* was lower than the small molecule mediated correction of class II CFTR mutations *in vitro* in transfected cell lines (Han et al., 2018).

Even though only four major bands are observed in the CFTR immunoblots (B, C*, <95 kDa, 240 kDa), we noticed that the band pattern detected with antibodies raised against CFTR is more similar between two paired samples (samples prior to and after ELX/TEZ/IVA treatment) from one patient while patterns obtained from different individuals have a distinguishable signature (Figure 2C; Supplementary Figure S2). We interpret the low molecular weight bands of <95 kDa as degradation products of CFTR (Ward and Kopito, 1994; Gentzsch et al., 2004; Swiatecka-Urban et al., 2005). The high molecular weight signals at approximately 240 kDa may represent multimeric protein complexes such as ubiquitinated CFTR (Ameen et al., 2007; McClure et al., 2016) or CFTR non-covalently or covalently linked with members of the p.Phe508del CFTR interactome (Pankow et al., 2015; Vinhoven et al., 2021). Alternatively, the 240 kDa band might correspond to rootletin which can also be detected by mAb596 raised against CFTR (Sato et al., 2021). The similarity between samples taken from one patient suggests that the repertoire of glycosylation enzymes, degradation enzymes and CFTR interacting partners is likely specific for an individual and thus leads to a unique set of CFTR glycoisoforms and CFTR multiprotein species in each patient.

Studies with recombinant CFTR in transfected cell lines have revealed that neither core nor complex N-glycans are required for the correct folding of CFTR at the ER and the subsequent trafficking to the cell surface (Chang et al., 2008). However, the N-glycans enhance the productive folding and conformational stability of CFTR (Glozman et al., 2009). Defective N-glycosylation reduces the stability of CFTR, induces ubiquitination and causes more rapid turnover in post-ER compartments (McClure et al., 2016). In our study, the pharmacologically rescued band C* p.Phe508del CFTR from patients’ rectal mucosa differed from the complex-glycosylated isoform C of wild-type CFTR by higher mean mobility and lower bandwidth of the immune-reactive signal on the Western blot (Figures 1, 2; Supplementary Figure S2). In the Golgi apparatus, complex glycan attachment by one of the more than 200 glycosyltransferases fine-tunes protein biogenesis (Lairson et al., 2008). The broad poly-disperse distribution of the wild type Golgi maximally tetra-antennary glycoform C of CFTR is most likely caused by repeating units of N-acetyllactosamine (O’Riordan et al., 2000). Hence, under the assumption that CFTR-C* is a partially glycosylated isoform of CFTR, it is conceivable that in the rectal biopsies we examined CFTR triple combination therapy promoted the exit of p.Phe508del CFTR from the ER (Kleizen et al., 2021), but failed to restore Golgi-resident glycosylation steps including the addition of N-acetyllactosamine repeats. Alternatively, as wild-type and mutant CFTR are modified differently post-translationally (Gong et al., 2019; Wu et al., 2022), the size difference between CFTR-B and CFTR-C* might be the result of other posttranslational modifications such as mono- or poly-ubiquinylation or sumoylation.

Any factors that influence the synthesis, processing, trafficking, half-life of mutant CFTR such as the members of the CFTR interactome (Pankow et al., 2015; Vinhoven et al., 2021) may be considered as the primary modifiers of cellular CFTR protein content. Alternative ion channels (Pinto et al., 2021) and the growth, differentiation, ageing and remodeling of tissue (Brezillon et al., 1995; Castillon et al., 2002) constitute the secondary modifiers of ion channel function; and lifestyle, living conditions, socioeconomic status, biological age, gender, therapeutic measures and co-morbidities represent the tertiary modifiers of CFTR homeostasis.

It has been noted in ELX/TEZ/IVA treated primary cultures of nasal and pulmonary epithelia that the functional correction exceeds the biochemical correction of CFTR (Veit et al., 2020), indicating that the potentiator function of ELX in these respiratory primary epithelia (Veit et al., 2020; Shaughnessy et al., 2021) can partially compensate for mistrafficking and inadequate processing of mutant CFTR. Since the degree of clinical benefit changes within a small range of functional CFTR protein, it remains to be seen whether the strong clinical benefit of the treatment with ELX/TEZ/IVA seen in our study and the clinical trials (Heijerman et al., 2019; Middleton et al., 2019) will persist or attenuate over the years in an ageing CF population. Our results might provide a basis to understand different degrees in response and different long-term outcomes of ELX/TEZ/IVA treatment. They caution that the lower amounts or immature glycosylation of the C* glycoisoform might prevent long-term, sustained benefit of ELX/TEZ/IVA. The development of further CFTR modulators that promote the production of more and mature CFTR may increase the robustness of the functional rescue and may reduce the strong patient-to-patient variation of the clinical response. Further analyses of CFTR glycoisoforms by high-resolution western blot from patient’s biosamples may thus assist to verify and monitor the individual’s CFTR-C glycosylation status achieved by different CFTR small molecule therapeutics as a potential biomarker for full functional CFTR correction. Given the clinical success of ELX/TEZ/IVA triple therapy, we hope that our results can provide leverage to achieve improved functional rescue of CFTR and provide guidance for the development of approaches to rescue protein function for other diseases affected by aberrations of protein synthesis, post-translational processing and trafficking.

## Data Availability

The original contributions presented in the study are included in the article/Supplementary Material, further inquiries can be directed to the corresponding author.
