# Supplementary material for: Changes in cystic fibrosis transmembrane conductance regulator protein expression prior to and during elexacaftor-tezacaftor-ivacaftor therapy
Source: Front Pharmacol. 2023 Jan 27;14:1114584. doi: 10.3389/fphar.2023.1114584 (PMC9911415; doi:10.3389/fphar.2023.1114584)
Supplement: Supplementary file 1 [file DataSheet2.PDF]

Supplementary Material

Changes in cystic fibrosis transmembrane conductance regulator protein expression prior to and during elexacaftor-tezacaftor-ivacaftor therapy

Frauke Stanke[1,2]*, Sophia T. Pallenberg[1], Stephanie Tamm[1,2], Silke Hedtfeld[1], Ella Marie Eichhorn[1], Rebecca Minso[1], Gesine Hansen[1,2], Tobias Welte[3,2], Annette Sauer-Heilborn[3], Felix C. Ringshausen[3,2], Sibylle Junge[1], Burkhard Tümmler[1,2]‡, Anna-Maria Dittrich[1,2]‡

[1]Department of Pediatric Pneumology, Allergology and Neonatology, Hannover Medical School, D-30625 Hannover, Germany

[2]Biomedical Research in Endstage and Obstructive Lung Disease Hannover (BREATH), German Center for Lung Research, Hannover Medical School, Hannover, Germany

[3]Department of Respiratory Medicine, Hannover Medical School, D-30625 Hannover, Germany

‡ BT and AMD contributed equally

*** Correspondence:**
PD Dr. rer. nat. Frauke Stanke
e-mail: mekus.frauke@mh-hannover.de
ORCID-ID 0000-0002-6186-0149

Source data for Figure 2 "Changes in CFTR glycoisoforms from rectal suction biopsies upon treatment with ELX/TEZ/IVA"

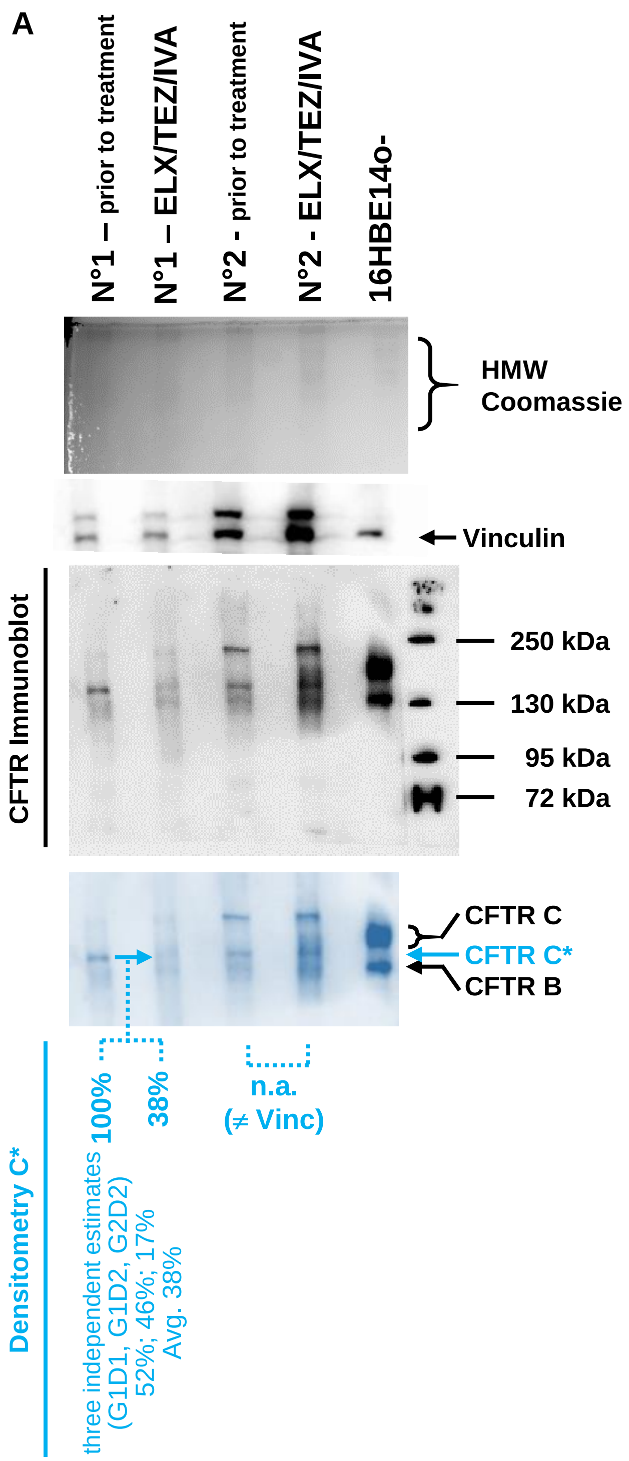
A
N°1 – prior to treatment
N°1 – ELX/TEZ/IVA
N°2 - prior to treatment
N°2 - ELX/TEZ/IVA
16HBE14o-
HMW
Coomassie
Vinculin
CFTR Immunoblot
250 kDa
130 kDa
95 kDa
72 kDa
CFTR C
CFTR C*
CFTR B
Densitometry C*
100%
38%
n.a.
(≠ Vinc)
three independent estimates
(G1D1, G1D2, G2D2)
52%; 46%; 17%
Avg. 38%

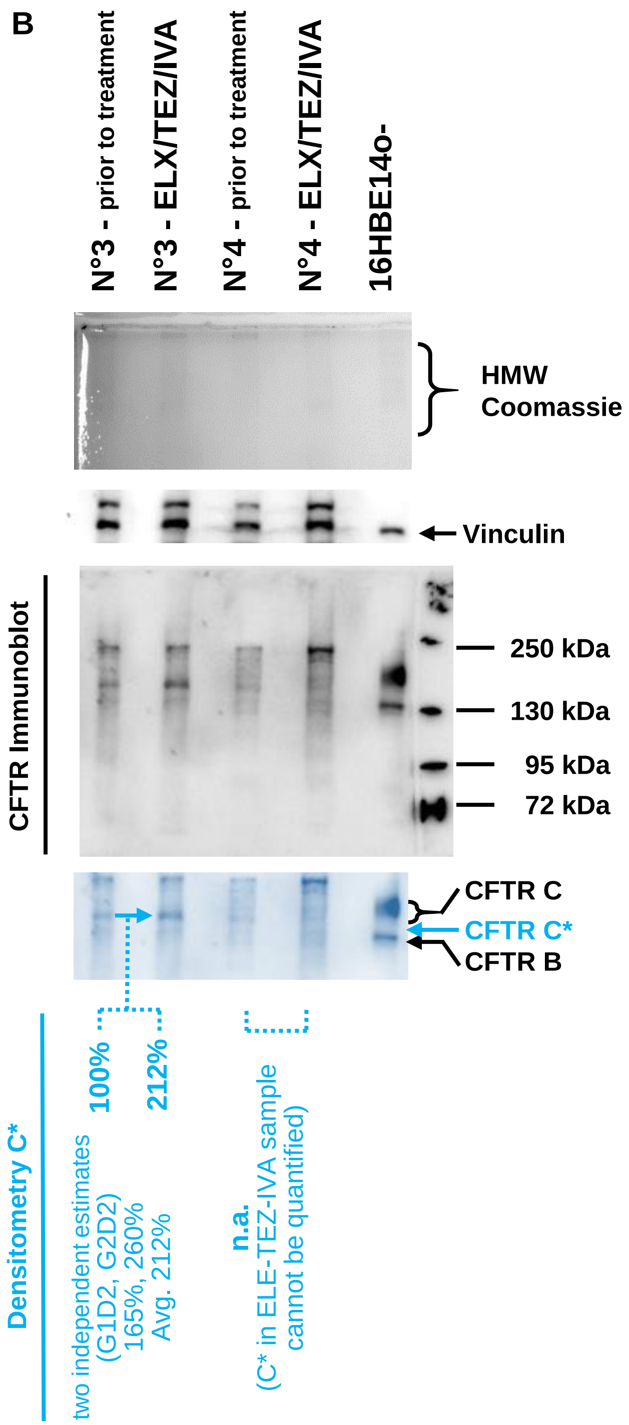
B
N°3 - prior to treatment
N°3 - ELX/TEZ/IVA
N°4 - prior to treatment
N°4 - ELX/TEZ/IVA
16HBE14o-
HMW
Coomassie
Vinculin
CFTR Immunoblot
250 kDa
130 kDa
95 kDa
72 kDa
CFTR C
CFTR C*
CFTR B
Densitometry C*
100%
212%
two independent estimates
(G1D2, G2D2)
165%, 260%
Avg. 212%
n.a.
(C* in ELE-TEZ-IVA sample
cannot be quantified)

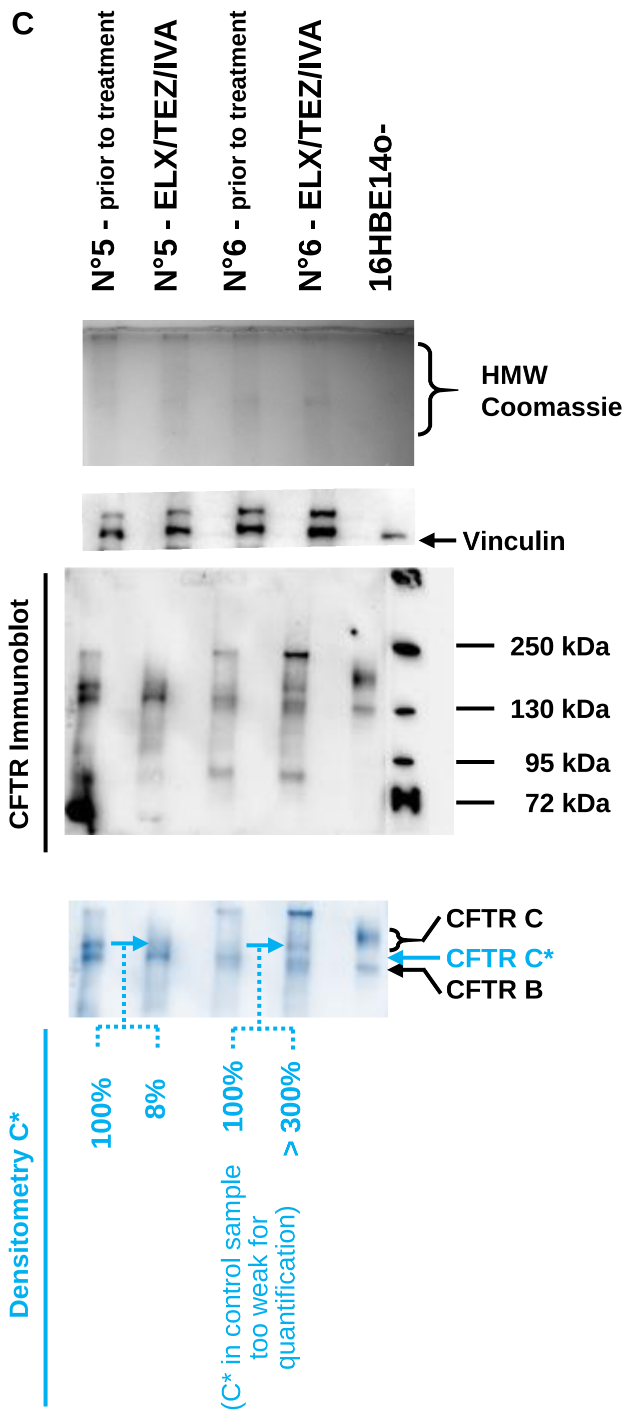
C
N°5 - prior to treatment
N°5 - ELX/TEZ/IVA
N°6 - prior to treatment
N°6 - ELX/TEZ/IVA
16HBE14o-
HMW Coomassie
Vinculin
CFTR Immunoblot
250 kDa
130 kDa
95 kDa
72 kDa
CFTR C
CFTR C*
CFTR B
Densitometry C*
100%
8%
100%
> 300%
(C* in control sample too weak for quantification)

D

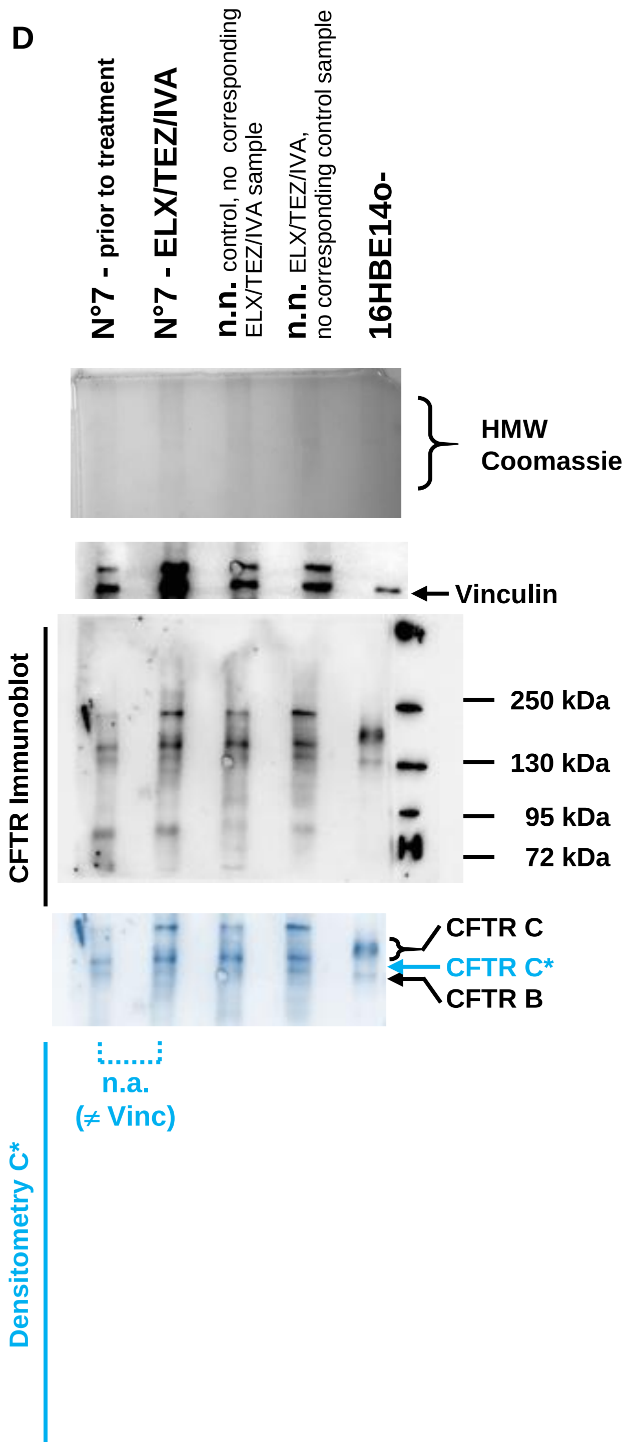
N°7 - prior to treatment
N°7 - ELX/TEZ/IVA
n.n. control, no corresponding ELX/TEZ/IVA sample
n.n. ELX/TEZ/IVA, no corresponding control sample
16HBE14o-
HMW Coomassie
Vinculin
CFTR Immunoblot
250 kDa
130 kDa
95 kDa
72 kDa
CFTR C
CFTR C*
CFTR B
n.a.
(≠ Vinc)
Densitometry C*

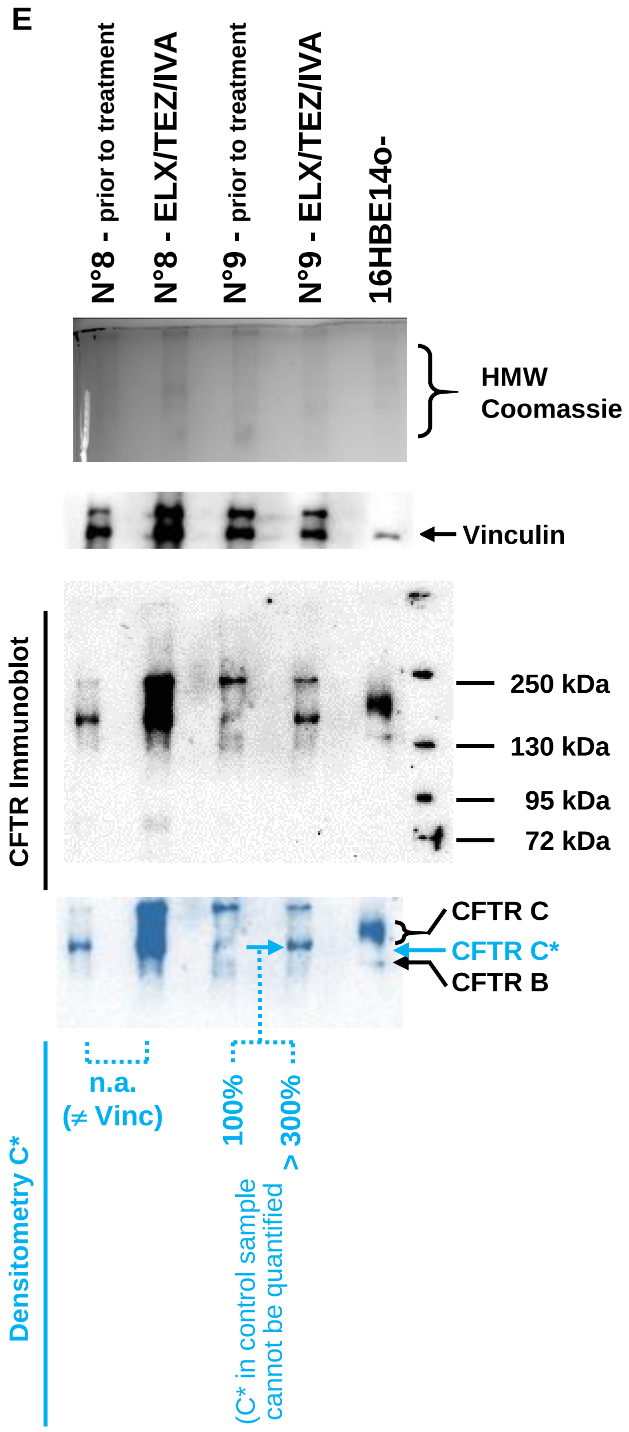
E
N°8 - prior to treatment
N°8 - ELX/TEZ/IVA
N°9 - prior to treatment
N°9 - ELX/TEZ/IVA
16HBE14o-
HMW
Coomassie
Vinculin
CFTR Immunoblot
250 kDa
130 kDa
95 kDa
72 kDa
CFTR C
CFTR C*
CFTR B
Densitometry C*
n.a.
(≠ Vinc)
100%
> 300%
(C* in control sample
cannot be quantified

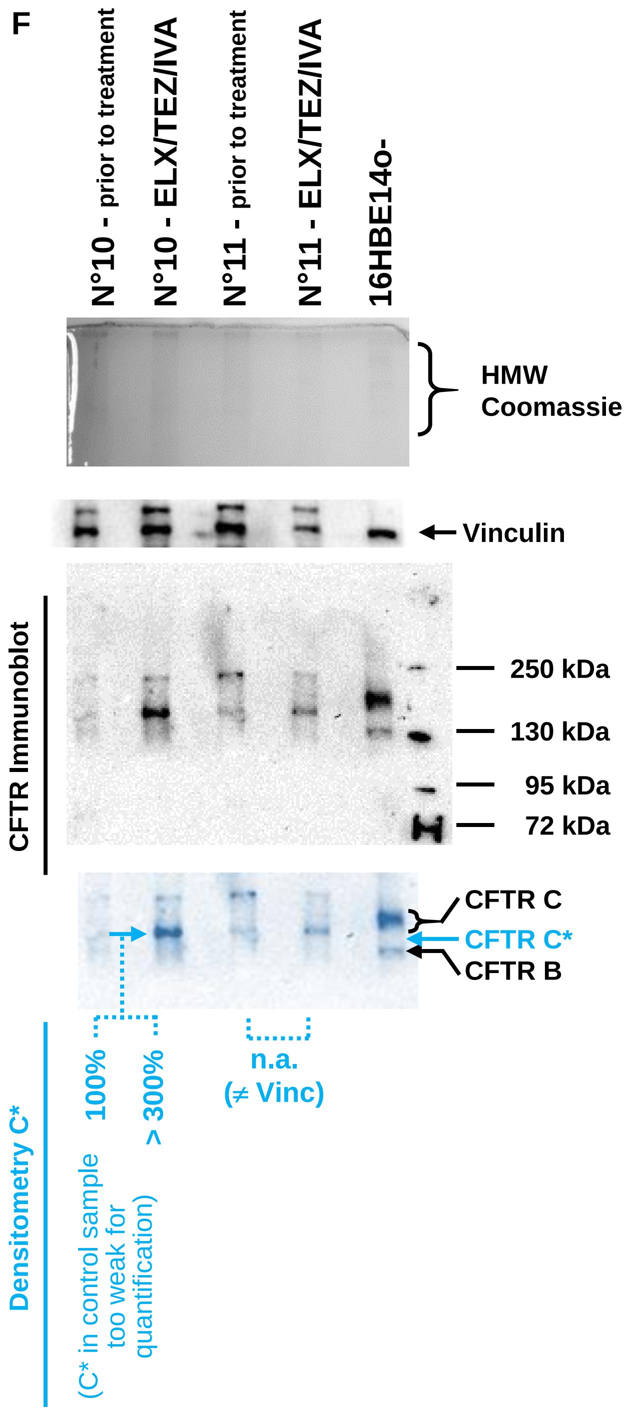
F
N°10 - prior to treatment
N°10 - ELX/TEZ/IVA
N°11 - prior to treatment
N°11 - ELX/TEZ/IVA
16HBE14o-
HMW Coomassie
Vinculin
CFTR Immunoblot
250 kDa
130 kDa
95 kDa
72 kDa
CFTR C
CFTR C*
CFTR B
Densitometry C*
100%
> 300%
n.a.
(≠ Vinc)
(C* in control sample too weak for quantification)

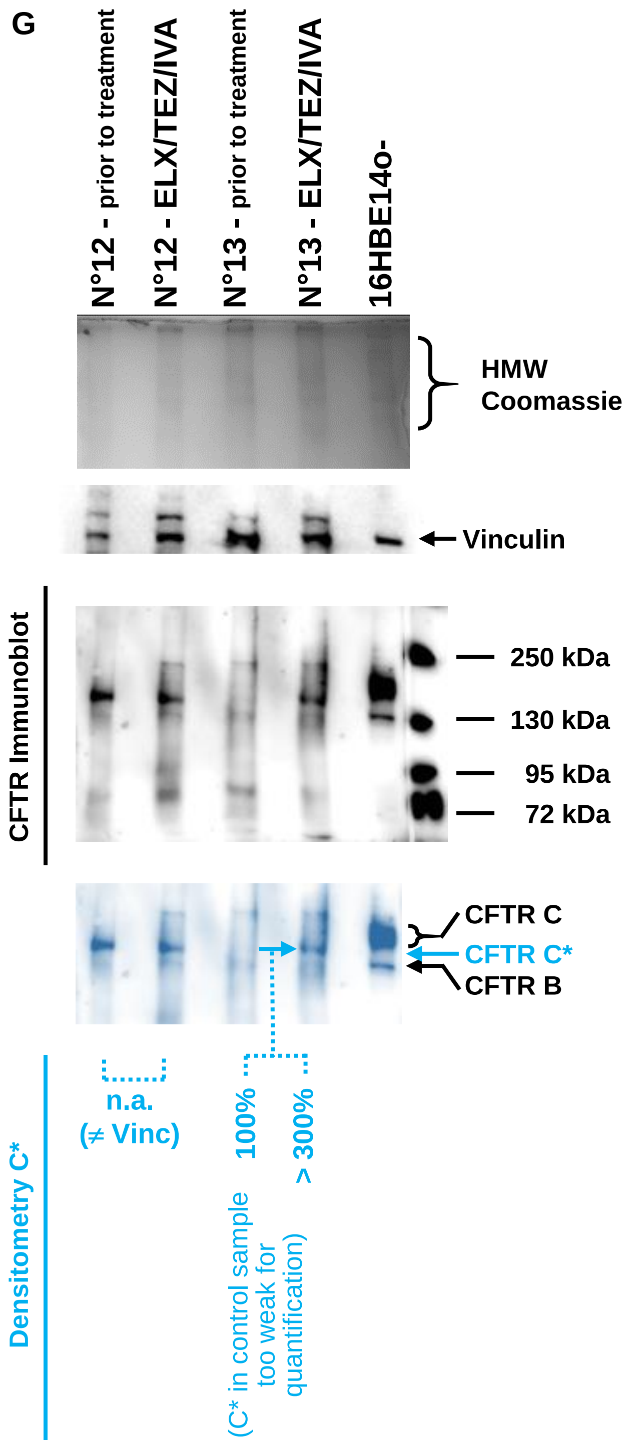
G
N°12 - prior to treatment
N°12 - ELX/TEZ/IVA
N°13 - prior to treatment
N°13 - ELX/TEZ/IVA
16HBE14o-
HMW
Coomassie
Vinculin
CFTR Immunoblot
250 kDa
130 kDa
95 kDa
72 kDa
CFTR C
CFTR C*
CFTR B
Densitometry C*
n.a.
(≠ Vinc)
100%
> 300%
(C* in control sample
too weak for
quantification)

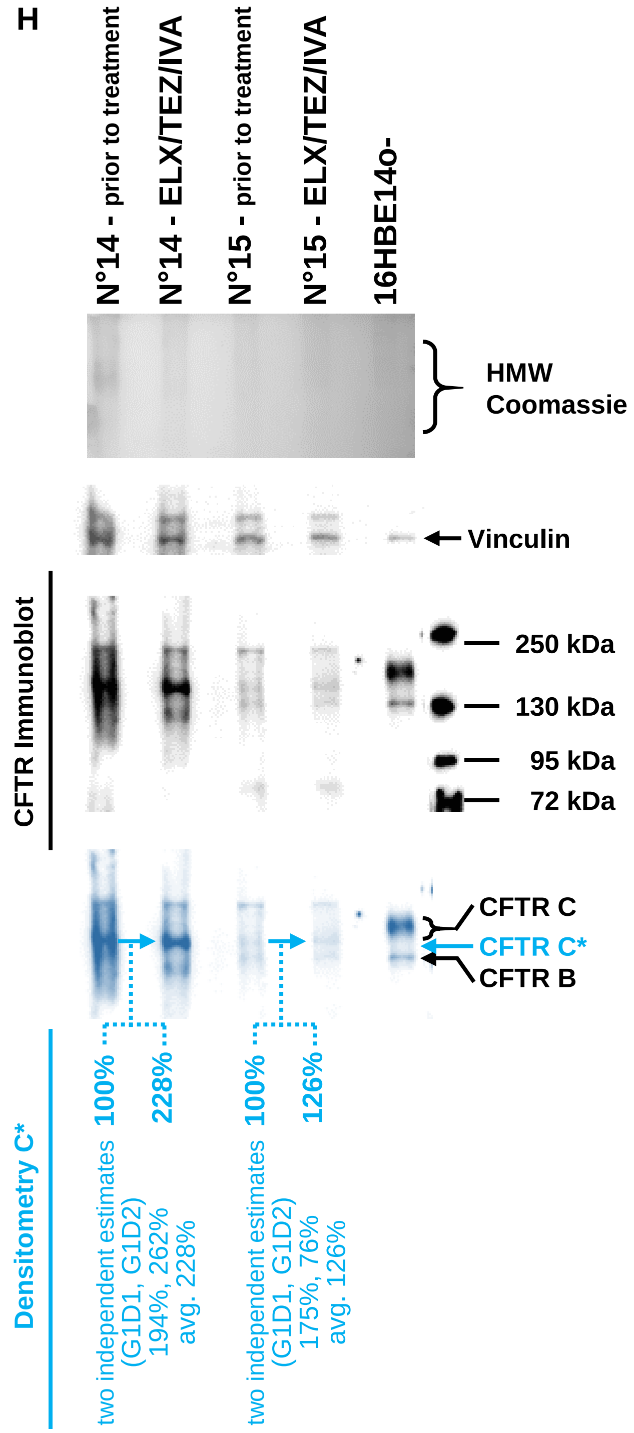
H
N°14 - prior to treatment
N°14 - ELX/TEZ/IVA
N°15 - prior to treatment
N°15 - ELX/TEZ/IVA
16HBE14o-
HMW
Coomassie
Vinculin
CFTR Immunoblot
250 kDa
130 kDa
95 kDa
72 kDa
CFTR C
CFTR C*
CFTR B
Densitometry C*
100%
228%
100%
126%
two independent estimates
(G1D1, G1D2)
194%, 262%
avg. 228%
two independent estimates
(G1D1, G1D2)
175%, 76%
avg. 126%

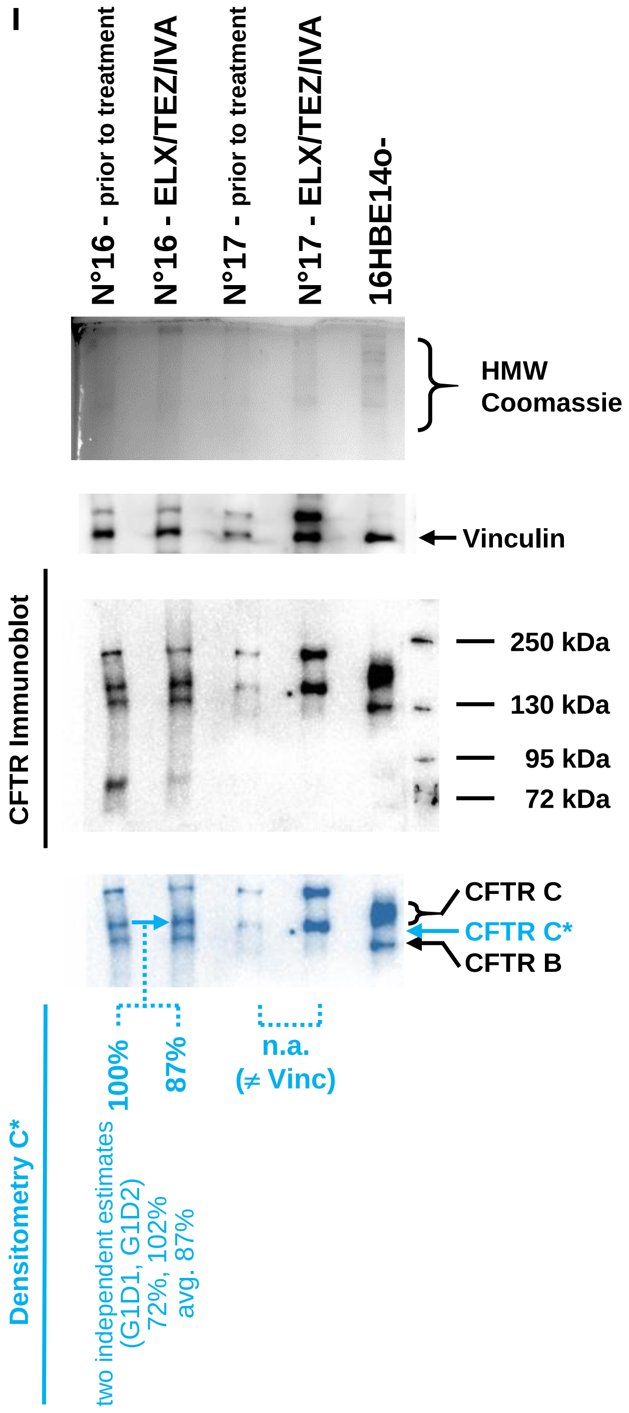
I
N°16 - prior to treatment
N°16 - ELX/TEZ/IVA
N°17 - prior to treatment
N°17 - ELX/TEZ/IVA
16HBE14o-
HMW Coomassie
Vinculin
CFTR Immunoblot
250 kDa
130 kDa
95 kDa
72 kDa
CFTR C
CFTR C*
CFTR B
100%
87%
n.a.
(≠ Vinc)
Densitometry C*
two independent estimates
(G1D1, G1D2)
72%, 102%
avg. 87%

J

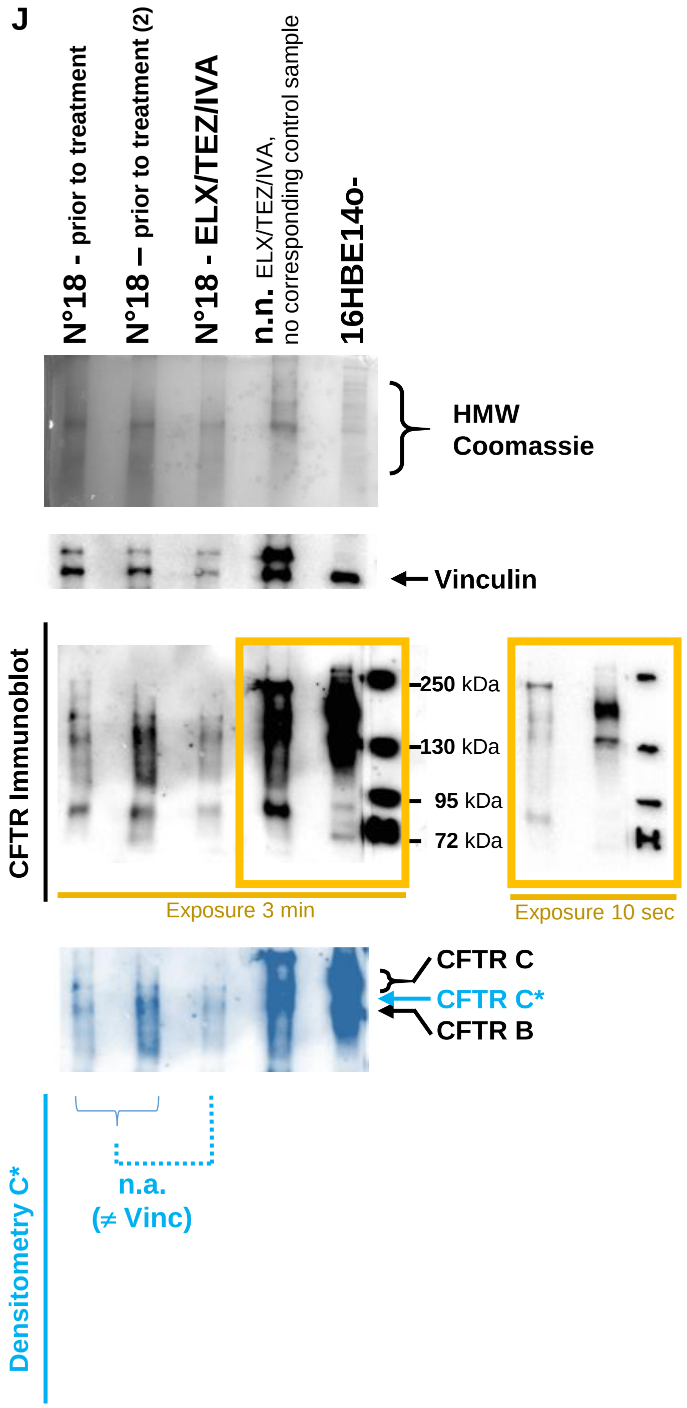
N°18 - prior to treatment
N°18 – prior to treatment (2)
N°18 - ELX/TEZ/IVA
n.n. ELX/TEZ/IVA, no corresponding control sample
16HBE14o-
HMW Coomassie
Vinculin
CFTR Immunoblot
250 kDa
130 kDa
95 kDa
72 kDa
Exposure 3 min
Exposure 10 sec
CFTR C
CFTR C*
CFTR B
Densitometry C*
n.a.
(≠ Vinc)

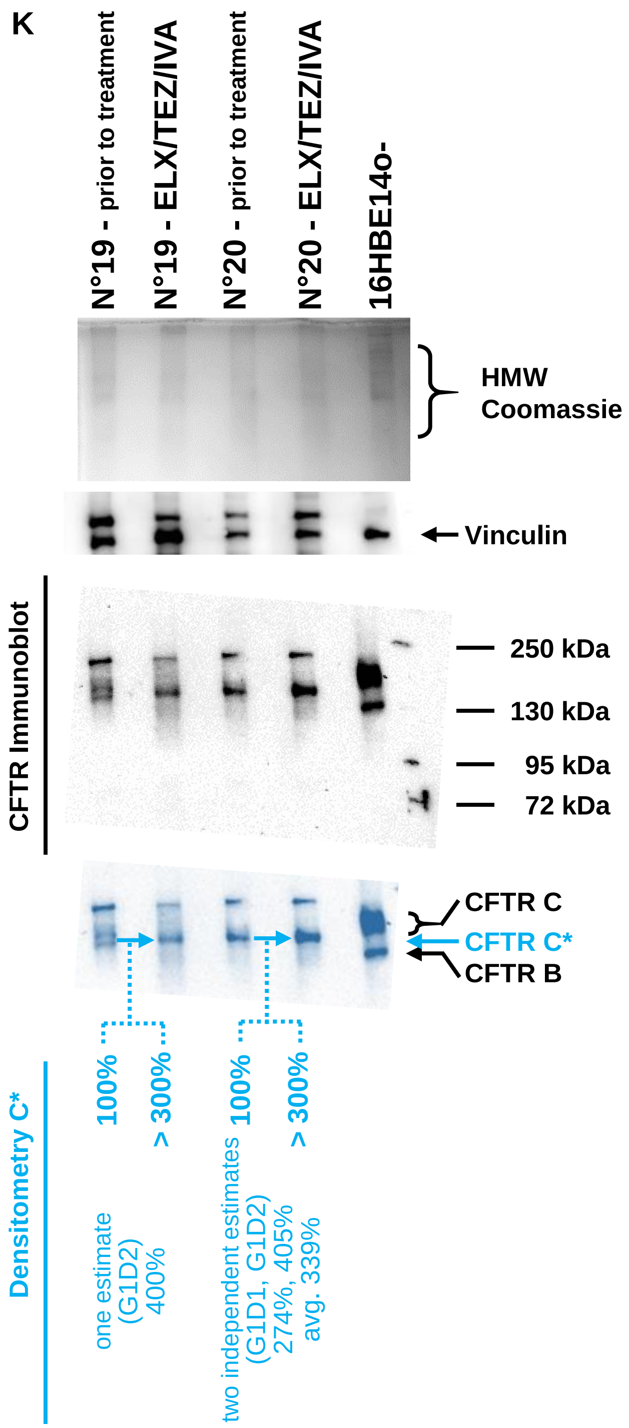
K
N°19 - prior to treatment
N°19 - ELX/TEZ/IVA
N°20 - prior to treatment
N°20 - ELX/TEZ/IVA
16HBE14o-
HMW
Coomassie
Vinculin
CFTR Immunoblot
250 kDa
130 kDa
95 kDa
72 kDa
CFTR C
CFTR C*
CFTR B
Densitometry C*
100%
> 300%
100%
> 300%
one estimate
(G1D2)
400%
two independent estimates
(G1D1, G1D2)
274%, 405%
avg. 339%

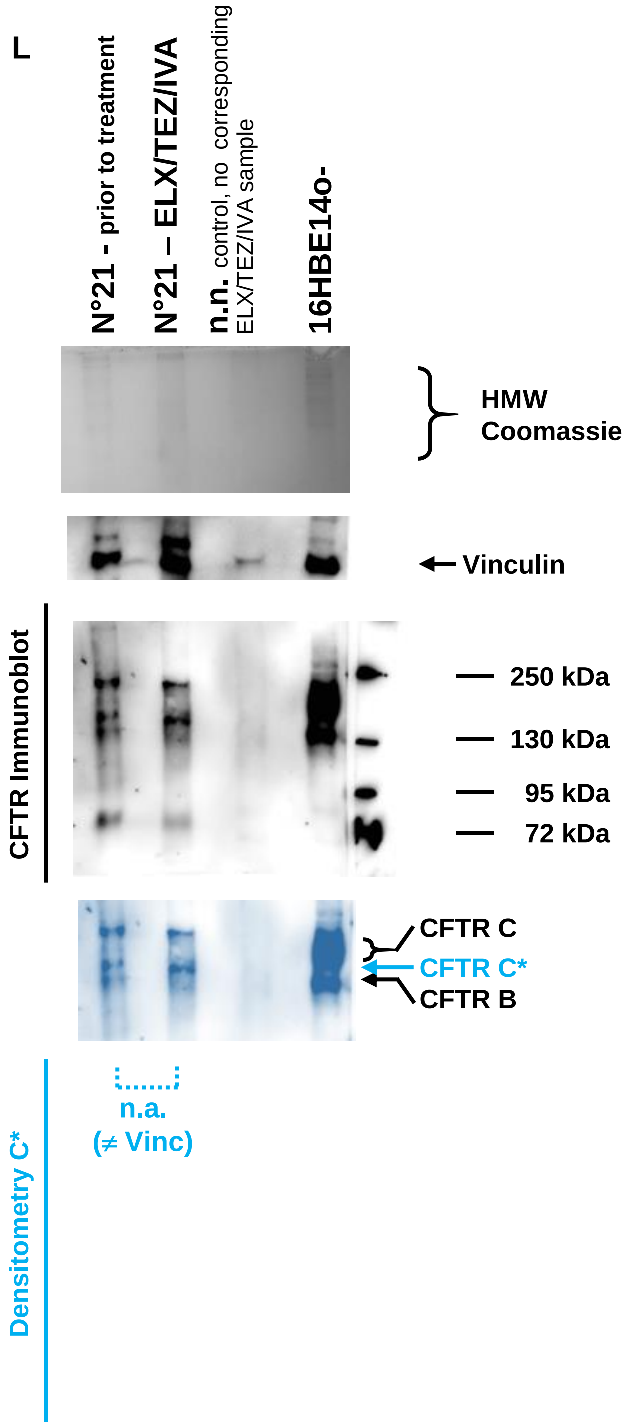

L
N°21 - prior to treatment
N°21 – ELX/TEZ/IVA
n.n. control, no corresponding ELX/TEZ/IVA sample
16HBE14o-
HMW Coomassie
Vinculin
CFTR Immunoblot
250 kDa
130 kDa
95 kDa
72 kDa
CFTR C
CFTR C*
CFTR B
n.a.
(≠ Vinc)
Densitometry C*

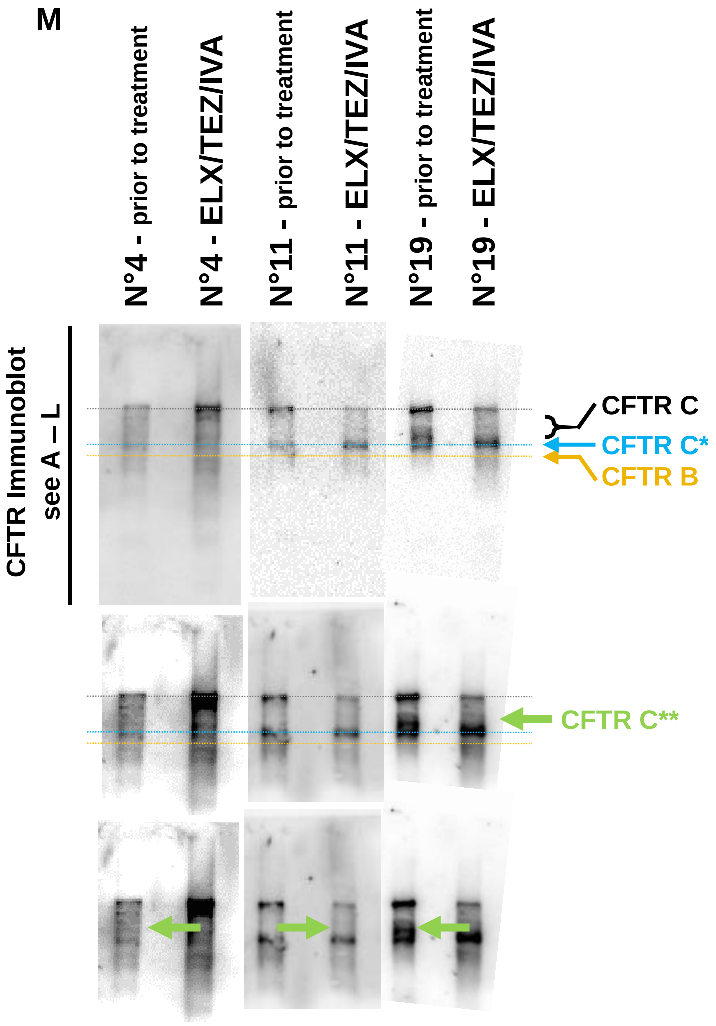
M
N°4 - prior to treatment
N°4 - ELX/TEZ/IVA
N°11 - prior to treatment
N°11 - ELX/TEZ/IVA
N°19 - prior to treatment
N°19 - ELX/TEZ/IVA
CFTR Immunoblot
see A – L
CFTR C
CFTR C*
CFTR B
CFTR C**

Supplementary Figure 2 A-M: Source data for Figure 2 "Changes in CFTR glycoisoforms from rectal suction biopsies upon treatment with ELX/TEZ/IVA"

A-L: CFTR Immunoblots prior to and after the start of ELX/TEZ/IVA treatment were obtained from rectal suction biopsies of 21 patients. Panel A to L show: loading control HMW Coomassie staining, Vinculin detection and CFTR immunoblot primary data, assignment of CFTR-C* and densitometry if sample pair is eligible for semi-quantitative analysis (see methods for details). Western blot loading control. We have judged paired biomaterials by two controls: Firstly, based on nonspecific protein staining using Coomassie of leftover material after transfer of whole-cell lysates to the membrane, we have verified that the amount of proteins capable to enter the polyacrylamide matrix is comparable between samples obtained pre-treatment and after ELX/TEZ/IVA treatment from one patient. Next, specific vinculin staining was used to judge whether the paired biopsy samples contain an equivalent amount of epithelium. Based on Coomassie and /or specific vinculin staining, we excluded 8 samples from further densitometric analysis of CFTR band C* since samples prior-to-treatment and ELX/TEZ/IVA sample were incomparable for these 8 pairs: N°2 – panel A, N°7 – panel D, N°8 – panel E, N°11 – panel F, N°12 – panel G, N°17 – panel I, N°18 panel J, N°21 – panel L. Densitometry confirms that censored samples differed stronger than samples included for densitometry: Signals for vinculin and metavinculin of censored 8 sample pairs differed by 1.3 fold (SD 1.6 fold) when comparing prior-to-treatment and TEZ-ELE-IVA-sample. Vinculin and metavinculin signals in 14 sample pairs accepted for densitometry differed by 0.4 fold (SD 0.2 fold) when comparing prior-to-treatment and ELX/TEZ/IVA lane. Semiquantitative analysis of band CFTR C*- technical replicates. For the remaining 13 samples, CFTR band C* was quantified by densitometry from both paired samples. If possible, we have evaluated more than one CFTR detection as indicated below the densitometry data in Figures A to L whereby G1D1, G1D2 and G2D2 (see below) were used to describe technical replicates in the source data. Respectively, samples were analyzed by gel electrophoresis, transferred to a membrane and subsequent detection of CFTR twice in sequence whereby the membrane was not stripped of antibodies between these two detection steps which were marked by G1D1 and G1D2 in panels A to L. For four samples, the resolution during gel electrophoresis was insufficient to recognize Band C CFTR in the 16HBE14o- control lane (samples N°1 to N°4), thus, these were repeated (panel A and panel B). Semi-quantitative expression data for sample 1 could be judged from three independent estimates G1D1, G1D2, G2D2. Semiquantitative analysis of band CFTR C*- weak signals of CFTR-C*. The intensity of CFTR band C* was minute in four control samples (N°6 – panel C, N°9 – panel E, N°10 – panel F, N°13 – panel G), making normalization to 100% as expression level prior to start of treatment error prone. Based on obtained primary data of samples N°19 and N°20 corresponding to an increase of CFTR C* of 400%, 274% and 405% (see panel K), we used a cut-off value of >300% gain in signal for band CFTR C* to describe the increase in CFTR expression. In sample pair N°4 (panel B), the low intensity band C* could not be quantified in the sample obtained after treatment with ELX/TEZ/IVA and thus change upon treatment was not quantified in this sample.

M: Further CFTR glycoisoforms than CFTR C*. We have carefully inspected the immunoblots for a CFTR isoform that has migratory properties between the mutant glycosylated CFTR band C* and the mature, wild-type CFTR seen in the 16HBE14o- control samples. In the three samples N°4, N°11 and N°19, such a band labelled as CFTR C** was observed (panel M). It is easily distinguishable from wild-type CFTR band C by firstly, migrating further into the gel indicating lower molecular weight, altered shape or altered charge/shape ratio and by secondly, being sharp and focused, indicative of low complexity and less variability in their N-glycans.
