# Supplementary material for: Changes in cystic fibrosis transmembrane conductance regulator protein expression prior to and during elexacaftor-tezacaftor-ivacaftor therapy
Source: Front Pharmacol. 2023 Jan 27;14:1114584. doi: 10.3389/fphar.2023.1114584 (PMC9911415; doi:10.3389/fphar.2023.1114584)
Supplement: Supplementary file 2 [file DataSheet3.PDF]

Supplementary Material

Changes in cystic fibrosis transmembrane conductance regulator protein expression prior to and during elexacaftor-tezacaftor-ivacaftor therapy

Frauke Stanke[1,2]*, Sophia T. Pallenberg[1], Stephanie Tamm[1,2], Silke Hedtfeld[1], Ella Marie Eichhorn[1], Rebecca Minso[1], Gesine Hansen[1,2], Tobias Welte[3,2], Annette Sauer-Heilborn[3], Felix C. Ringshausen[3,2], Sibylle Junge[1], Burkhard Tümmler[1,2]‡, Anna-Maria Dittrich[1,2]‡

[1]Department of Pediatric Pneumology, Allergology and Neonatology, Hannover Medical School, D-30625 Hannover, Germany

[2]Biomedical Research in Endstage and Obstructive Lung Disease Hannover (BREATH), German Center for Lung Research, Hannover Medical School, Hannover, Germany

[3]Department of Respiratory Medicine, Hannover Medical School, D-30625 Hannover, Germany

‡ BT and AMD contributed equally

*** Correspondence:**
PD Dr. rer. nat. Frauke Stanke
e-mail: mekus.frauke@mh-hannover.de
ORCID-ID 0000-0002-6186-0149

Supplementary Figure 3 for "CFTR immunoblot analysis of rectal mucosa from wild-type control biomaterials and CF patients who participate in the ELX/TEZ/IVA study."

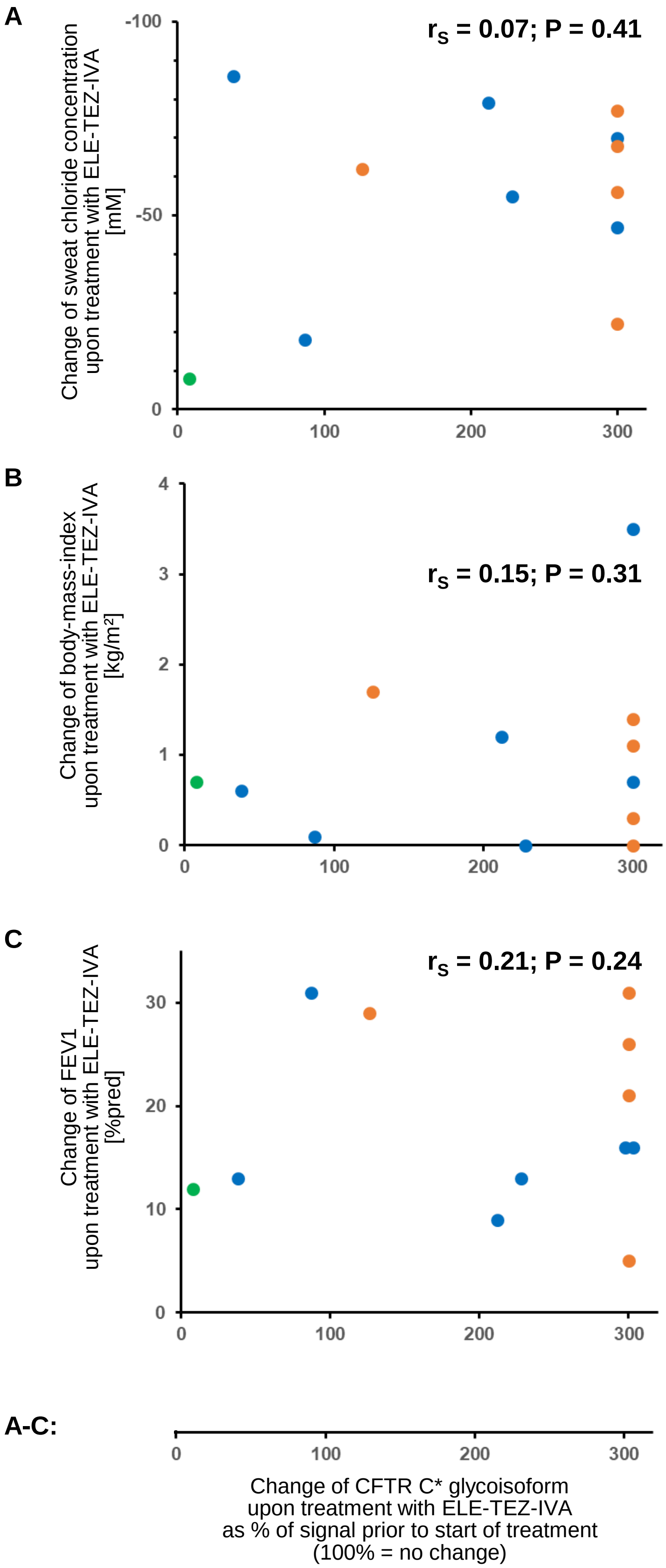
A
r_S = 0.07; P = 0.41
Change of sweat chloride concentration upon treatment with ELE-TEZ-IVA [mM]
-100
-50
0
0
100
200
300
B
r_S = 0.15; P = 0.31
Change of body-mass-index upon treatment with ELE-TEZ-IVA [kg/m²]
4
3
2
1
0
0
100
200
300
C
r_S = 0.21; P = 0.24
Change of FEV1 upon treatment with ELE-TEZ-IVA [%pred]
30
20
10
0
0
100
200
300
A-C:
0
100
200
300
Change of CFTR C* glycoisoform
upon treatment with ELE-TEZ-IVA
as % of signal prior to start of treatment
(100% = no change)

Supplementary Figure 3: Comparison of change in mutant glycoisoform CFTR-C* expression to change in clinical parameters upon treatment with ELE-TEZ-IVA

The change in protein expression of the mutant glycoisoform (see Figure 2) CFTR-C* was compared to the change in sweat chloride (**A**), body-mass-index (**B**) and lung function (**C**) (see Table 1) upon treatment with ELE-TEZ-IVA. Kindly note that while an improvement of clinical parameters was observed for all patients, CFTR-C* expression was not predictive for either change in sweat chloride nor BMI nor lung function. This could be due to the low power of the correlation analysis given our small sample size and limited sensitivity as for five samples indicated at 300% change of CFTR-C* expression, we could not quantify the increase of CFTR-C* expression precisely as prior to the start of ELE-TEZ-IVA treatment, CFTR-C* expression was too low for densitometry in these patients. **(A-C):** The patient's CFTR mutation genotype is colour-coded as follows: p.Phe508del homozygotes – terracotta; p.Phe508del compound heterozygotes with a class I mutation – blue; p.Phe508del / N1303K compound heterozygote – green.
