# Supplementary material for: Changes in cystic fibrosis transmembrane conductance regulator protein expression prior to and during elexacaftor-tezacaftor-ivacaftor therapy
Source: Front Pharmacol. 2023 Jan 27;14:1114584. doi: 10.3389/fphar.2023.1114584 (PMC9911415; doi:10.3389/fphar.2023.1114584)
Supplement: Supplementary file 3 [file DataSheet1.PDF]

frontiers

Supplementary Material

Changes in cystic fibrosis transmembrane conductance regulator protein expression prior to and during elexacaftor-tezacaftor-ivacaftor therapy

Frauke Stanke[1,2]*, Sophia T. Pallenberg[1], Stephanie Tamm[1,2], Silke Hedtfeld[1], Ella Marie Eichhorn[1], Rebecca Minso[1], Gesine Hansen[1,2], Tobias Welte[3,2], Annette Sauer-Heilborn[3], Felix C. Ringshausen[3,2], Sibylle Junge[1], Burkhard Tümmler[1,2]‡, Anna-Maria Dittrich[1,2]‡

[1]Department of Pediatric Pneumology, Allergology and Neonatology, Hannover Medical School, D-30625 Hannover, Germany

[2]Biomedical Research in Endstage and Obstructive Lung Disease Hannover (BREATH), German Center for Lung Research, Hannover Medical School, Hannover, Germany

[3]Department of Respiratory Medicine, Hannover Medical School, D-30625 Hannover, Germany

‡ BT and AMD contributed equally

*** Correspondence:**
PD Dr. rer. nat. Frauke Stanke
e-mail: mekus.frauke@mh-hannover.de
ORCID-ID 0000-0002-6186-0149

Source data for Figure 1 "CFTR immunoblot analysis of rectal mucosa from wild-type control biomaterials and CF patients who participate in the ELX/TEZ/IVA study."

Fig. 1: CFTR Immunoblot

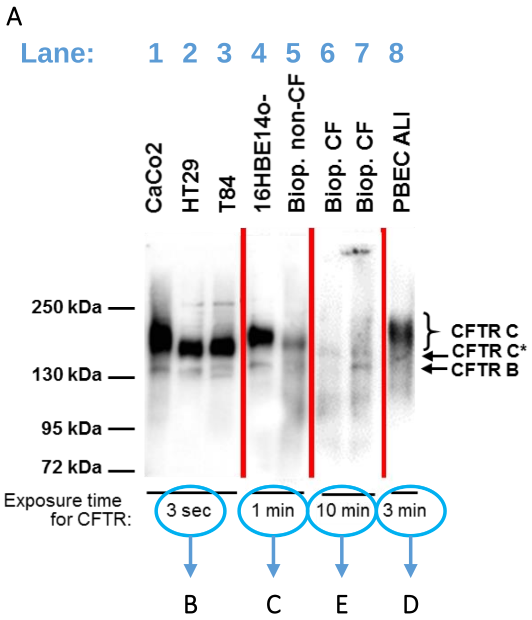

Source data CFTR Immunoblot

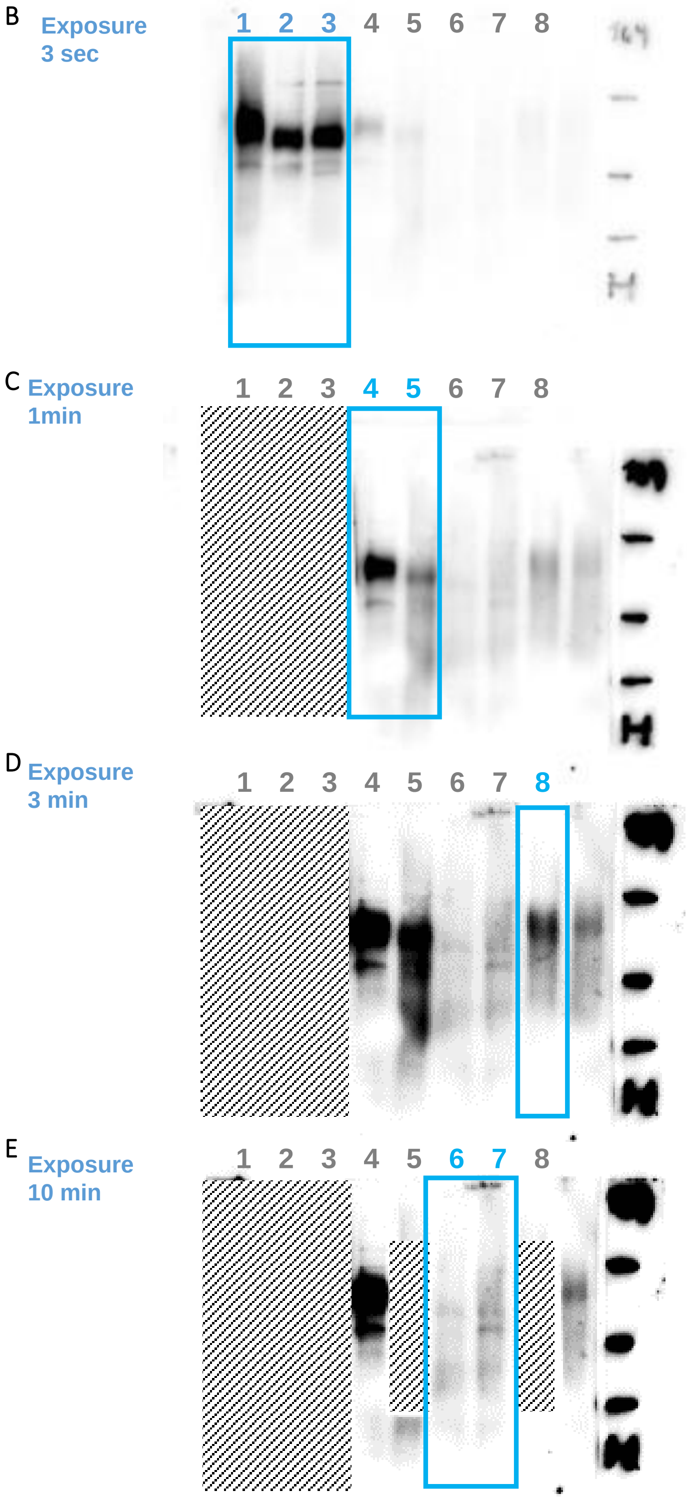

Supplementary Figure 1: Source data for Figure 1 “CFTR immunoblot analysis of rectal mucosa from wild-type control biomaterials and CF patients who participate in the ELX/TEZ/IVA study.”

The figure shown in the manuscript is derived from a single gel and a single western-blot membrane prepared thereof. While the high molecular weight bands stained with Coomassie (indicative of loaded protein amount that can enter the polyacrylamide gel) and signals for vinculin (marker for epithelial cells) can be derived from a single exposure, the CFTR amounts varies considerably between sources. Dynamics of the non-radioactive detection with the horseradish peroxidase substrate SuperSignal West Femto (Thermo Fisher Scientific) was feasible for all samples; however, different exposure times had to be chosen for visualization as indicated by blue rectangles in the source data. A: Immunoblot composite as provided for Figure 1. Four different exposures have been used to visualize all samples as indicated below with “B” to “E”. B –E: individual exposures for 3sec up to 10 min as indicated. Blue rectangles designate the area that is shown in figure 1. Areas marked by grey stripes were covered prior to detection to avoid that stronger signals from adjacent lanes obscure the weaker signals. Please note that CFTR-C*, visible in lane 6 and 7 in exposures C-E, migrates further (lower molecular weight or higher migratory properties) in comparison to the signal CFTR-C in lane 5 in exposure C.
